# The long-term and short-term effects of ambient air pollutants on sleep characteristics in the Chinese population: big data analysis from real world by sleep records of consumer wearable devices

**DOI:** 10.1186/s12916-023-02801-1

**Published:** 2023-03-08

**Authors:** Peining Zhou, Jing Ma, Xueying Li, Yixue Zhao, Kunyao Yu, Rui Su, Rui Zhou, Hui Wang, Guangfa Wang

**Affiliations:** 1 Department of Respiratory and Critical Care Medicine, Peking University First Hospital, 8 Xishiku Street, Xicheng District, Beijing, 100034 China; 2 Department of Medical Statistics, Peking University First Hospital, Beijing, China; 3 Zepp Health Corp., Hefei, China; 4 Bigdata and Cloud Platform BU, Zepp Health Corp., Hefei, China

**Keywords:** Air pollution, Big data, Sleep, Wearable device

## Abstract

Several studies on long-term air pollution exposure and sleep have reported inconsistent results. Large-scale studies on short-term air pollution exposures and sleep have not been conducted. We investigated the associations of long- and short-term exposure to ambient air pollutants with sleep in a Chinese population based on over 1 million nights of sleep data from consumer wearable devices. Air pollution data including particulate matter (PM_2.5_, PM_10_), nitrogen dioxide (NO_2_), sulfur dioxide (SO_2_), carbon monoxide (CO), and ozone (O_3_) were collected from the Ministry of Ecology and Environment. Short-term exposure was defined as a moving average of the exposure level for different lag days from Lag0 to Lag0-6. A 365-day moving average of air pollution was regarded as long-term exposure. Sleep data were recorded using wearable devices from 2017 to 2019. The mixed-effects model was used to evaluate the associations. We observed that sleep parameters were associated with long-term exposure to all air pollutants. Higher levels of air pollutant concentrations were associated with longer total sleep and light sleep duration, shorter deep sleep duration, and decreases in wake after sleep onset (WASO), with stronger associations of exposures to NO_2_ and CO [a 1-interquartile range (IQR) increased NO_2_ (10.3 μg/m^3^) was associated with 8.7 min (95% CI: 8.08 to 9.32) longer sleep duration, a 1-IQR increased CO (0.3 mg/m^3^) was associated with 5.0 min (95% CI: − 5.13 to − 4.89) shorter deep sleep duration, 7.7 min (95% CI: 7.46 to 7.85) longer light sleep duration, and 0.5% (95% CI: − 0.5 to − 0.4%) lower proportion of WASO duration to total sleep]. The cumulative effect of short-term exposure on Lag0-6 is similar to long-term exposure but relatively less. Subgroup analyses indicated generally greater effects on individuals who were female, younger (< 45 years), slept longer (≥ 7 h), and during cold seasons, but the pattern of effects was mixed. We supplemented two additional types of stratified analyses to reduce repeated measures of outcomes and exposures while accounting for individual variation. The results were consistent with the overall results, proving the robustness of the overall results. In summary, both short- and long-term exposure to air pollution affect sleep, and the effects are comparable. Although people tend to have prolonged total sleep duration with increasing air pollutant concentrations, their sleep quality might remain poor because of the reduction in deep sleep.

**Supplementary Information:**

The online version contains supplementary material available at 10.1186/s12916-023-02801-1.

## Introduction

Sleep is an important factor affecting health, similar to exercise and diet [1]. High-quality sleep is crucial for maintaining health and quality of life. Sleep disturbances have risen to be one of the major public health concerns.

Sleep disturbances are associated with numerous health problems such as cardiovascular events, diabetes, mental disorders, and cancer [2]. Previous publications [3, 4] have demonstrated the relationship between sleep duration and mortality using a U- shaped curve, whereby both short (< 7 h) and long (> 9 h) sleep duration could increase mortality risk, particularly in Asian populations [4]. Furthermore, sleep stability is potentially modifiable risk factors for cardiometabolic diseases. Decreased inter-daily stability increases hypertension prevalence and blood pressure [5]. Increased night-to-night sleep variability has been associated with an increased risk of adiposity, metabolic syndrome, and type 2 diabetes [6].

Numerous factors influence sleep quality, such as age, sex, physical activity, psychological or physiological conditions, and environmental factors [7]. Air pollution, another major public health concern, has been reported to affect sleep and has similar consequences to other diseases, such as cardiopulmonary health [8], diabetes [9], and cancer [10]. In particular, emerging research has recently focused on the effects of outdoor air pollution on sleep, as 91% of the worldwide population lives in places where the World Health Organization (WHO) ambient air quality guideline levels are not met [11].

Nevertheless, the relationship between ambient air pollution and sleep quality remains ambiguous and inconsistent. Many studies using questionnaires have revealed that poor air quality is associated with poor sleep quality [12]. Increased particulate matter with a diameter of 2.5 μm or less (PM_2.5_), particulate matter with a diameter of 10 μm or less (PM_10_), and nitrogen dioxide (NO_2_) concentrations are correlated with a reduction in daily sleep hours among college freshmen [13]. However, other studies have reported that air pollution deterioration is associated with increased sleep duration [14, 15] and wake times during sleep [16]. Long-term exposure to black carbon may induce shorter sleep duration in men and those with low socioeconomic status but longer sleep duration in blacks [17].

These discrepancies may result from different populations, study designs, pollutants, and, more importantly, methodologies of sleep evaluation. Almost no large-sample studies have employed objective sleep-scoring systems. Instead, most researchers have used a self-reported questionnaire or the Pittsburgh Sleep Quality questionnaire. The questionnaire tools will introduce bias due to their limitations and the participants’ cognition. With technological innovation, wearable devices, such as bracelets or watches, have owned the function to record and monitor wake or sleep in different stages [18–20], thus providing an excellent and convenient methodology for sleep evaluation. We analyzed a total of 1,245,817 nights of sleep records from a type of consumer bracelet in China between 2017 and 2019 and controlled several common influencing factors of sleep and air quality to clarify the long- and short-term effects of ambient air pollution on sleep.

## Methods

### Study population

A retrospective analysis was performed using data from consumer bracelets (Zepp Health Corp.) in China between 2017 and 2019. They were collected in an anonymous and aggregated dataset without personal identifiers such as names, email addresses, and cell phone numbers. Random strings were used to identify the sleep records for each night. The study was approved by the IRB of the Peking University First Hospital (2020-635).

In the real world, users often wear bracelets intermittently and irregularly, particularly during sleep. Only few people can wear bracelets continuously over a long period as the air quality fluctuates. For the study population, most people resided in a relatively fixed community, and only a small portion migrated or traveled frequently. Therefore, considering the privacy policy, we took each night’s record as a research object and used the air quality data collected at the sleep tracking site for lag analysis. We analyzed 1,245,817 nights of sleep data from 7682 participants for 3 years.

### Covariates

Several factors could influence sleep and were controlled in the statistical analysis, including registered sex, age, body mass index (BMI), city development level, altitude, season, and the type of night in which sleep records were tracked. The cities (five tiers) were classified based on development level according to business resource concentration, pivot function, the activity of urban residents, lifestyle diversity, and future plasticity, which has been widely quoted in China [21]. The sleep tracking seasons were divided into quarters in this study because of the large latitude span in China. Generally, in most parts of China, the first quarter (January to March) includes part of winter and early spring, the second quarter (April to June) includes spring and early summer, the third quarter (July to September) includes summer and early autumn, and the fourth quarter (October to December) includes the majority of autumn and winter. Additionally, we defined two types of night recordings: weeknight (Sunday to Thursday, the last night of the legal holidays) and night of rest (Friday, Saturday, the day before the legal holidays to the penultimate night).

### Data cleaning

We eliminated unreasonable or extreme values according to the following criteria to obtain eligible records from the raw data: registered age < 14 years, registered BMI < 15 kg/m^2^ or ≥ 45 kg/m^2^, total sleep duration ≤ 180 min or ≥ 720 min, mean heart rate of 24 h, or mean heart rate during sleep > 120 bpm.

### Sleep parameters

Sleep parameters recorded by the bracelets included total sleep duration (minutes of sleep per night for each participant), deep sleep duration, light sleep duration, times of wake after sleep onset (WASO), and duration of WASO. We used several ratios in the analysis to reduce the influence of total sleep duration on sleep parameters, such as deep sleep duration/total sleep duration, deep sleep duration/light sleep duration, times of WASO per hour of sleep, and durations of WASO per hour of sleep.

### Ambient air pollution data

The origin data of the main pollutants were collected from the National Urban Air Quality Real-time Publishing Platform (http://106.37.208.233:20035), linked to the open website of the Ministry of Ecology and Environment of the People’s Republic of China (https://www.mee.gov.cn/hjzl/). This website has been closed recently, and the corresponding data have been updated to a new website (http://air.cnemc.cn:18007/). These data were collected and reported every hour. This study measured the effect of short-term exposure with different lag days from Lag0 (record day) to Lag0-6. For instance, Lag0–6 represents the 7-day moving average of air pollutant concentrations between the record day and the 6th day before the record. Lag0-364 calculated a total of 365-day moving averages of air pollutant concentrations between the record day and the 364th day before the record, representing long-term exposure. The period of long-term exposure data for all participants ranged from 3 years (2016–2018). The data for PM_2.5_, PM_10,_ NO_2_, sulfur dioxide (SO_2_), and carbon monoxide (CO) were calculated from the mean estimated 24 h concentrations, and ozone (O_3_) was calculated from the maximum 8-h mean values. In addition, we matched the participants’ residential cities with the air pollution exposure data of the corresponding cities on the above website.

### Statistical analysis

We assessed normality and described distributions as mean, standard deviation (SD), minimum, and maximum for continuous variables or proportions for categorical variables. Mixed-effects model analysis was performed to investigate the associations of sleep parameters with ambient air pollution on both short- and long-term exposures because it allows the analysis of data from multiple measurements in one participant. Considering the high or moderate correlations among air pollutants (Supplementary Table S1), only single-pollutant models were used in our study to avoid collinearity.

The effect estimates were expressed as the change in sleep parameters per 1-IQR increase in each air-pollutant concentration with a random effect for each participant and fixed linear effects for air pollution and other covariates. Air pollutants were entered separately into single-pollutant models. The mixed-effects model was constructed using Eq.

$${Y}_{ij}={\beta}_0+{\beta}_{0\textrm{j}}+{\beta}_1{X}_{0 ij}\kern0.5em +{\beta}_2{X}_{1\textrm{ij}}\kern0.5em +{\beta}_3{X}_{2\textrm{ij}}\kern0.5em +{\beta}_4{X}_{3\textrm{ij}}+\dots \dots {\beta}_N{X}_{nij}+{\varepsilon}_{ij};$$

where *Y*_*ij*_ represents the sleep parameters, *β*_0_ is the fixed-effect intercept term, *β*_0*j*_ is the random-effect intercept term, *X*_0*ij*_ represents each air pollutant concentration, *β*_1_ is the regression coefficient for air pollutants, *β*2…*β*_N_ are the regression coefficients for the covariates in the model, *j* represents the study participant, *i* identifies the sleep record, and *ε*_*ij*_ is the residual error term. The results were presented as regression coefficients and 95% confidence intervals (CI). Additionally, the model was adjusted for other covariates, as noted previously. Subgroup analyses were conducted according to sex, age, season, and sleep duration. A cross-product term was added to the mixed-effects model to assess the significance of the interaction.

**Fig. 1:**
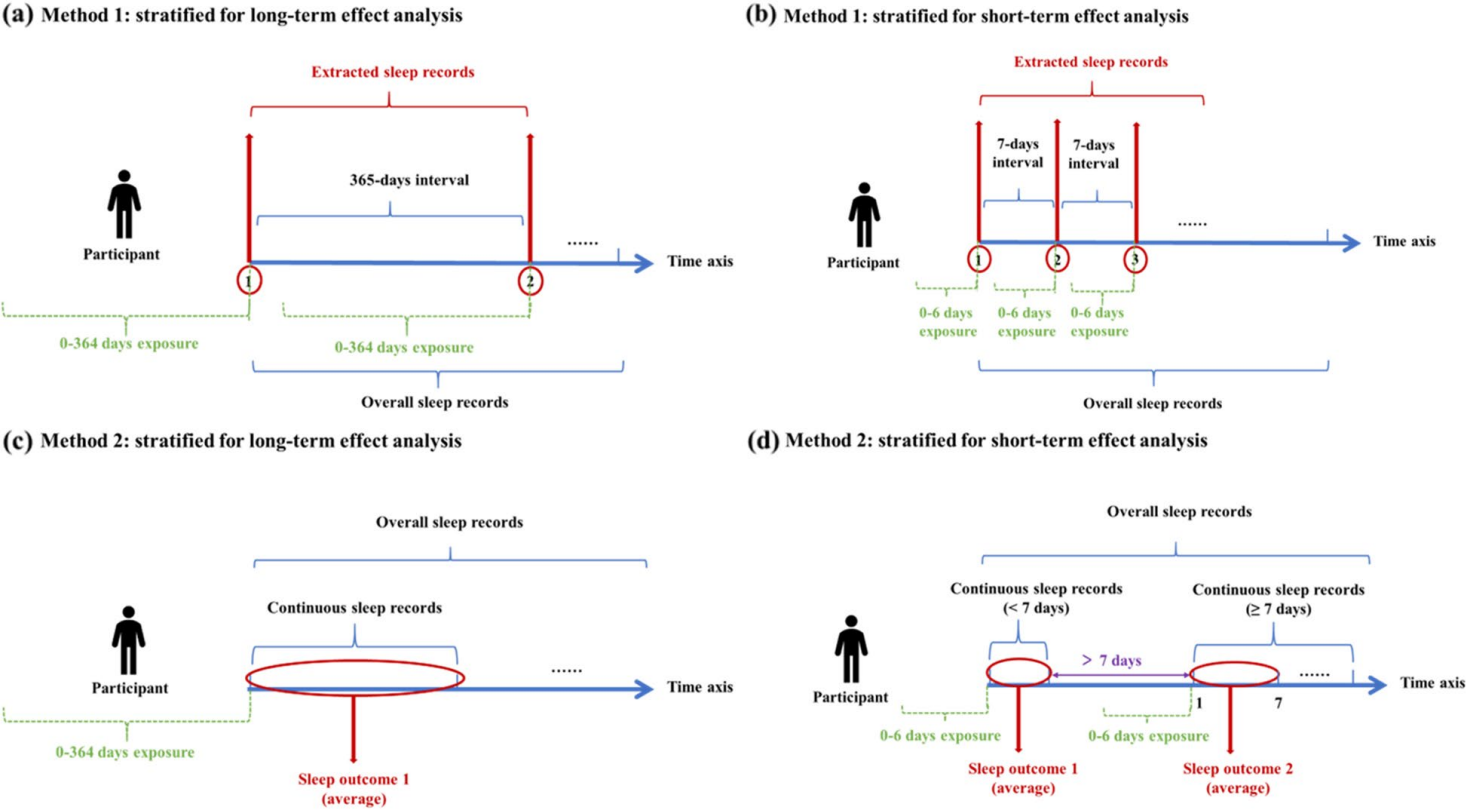
Two methods for stratified analyses

Given the bias caused by repeated measures of pollutant exposures and sleep parameters under the existing data structure, we further designed two stratified analyses to reduce repeated measures and consider individual variation, while still using the mixed-effects model. Figure 1 shows the two methods of stratified analysis. On the one hand, the sleep data for each participant were arranged in ascending chronological order. Then, starting from the first data of each subject, a piece of record was extracted every 7 days and 365 days intervals to analyze the impact of short- and long-term exposure on sleep. Alternatively, we regarded each continuous sleep record of each subject as a dataset and averaged the sleep parameters of each dataset for the analysis of long- and short-term effects. For the analysis of long-term effects, we averaged the sleep parameters of each subject’s first dataset and calculated air pollutant exposure based on the time of the first record in the dataset. Ultimately, only one piece of data was collected for each participant. For the analysis of short-term effects, we averaged the sleep parameters of the first 7 days of each dataset for each subject if the consecutive days of the dataset were ≥ 7 days. The time interval between the first record of each dataset and the last record of the previous valid dataset exceeded 7 days. If the consecutive days of the dataset were fewer than 7 days, the average of all sleep parameters in the dataset was calculated. Through these two stratified analyses, we sufficiently reduced the repeated measures of outcomes and exposures and took individual variation into account by calculating the mean value of sleep parameters, thus further verifying the stability of the overall data results.

Analyses were conducted using the SPSS statistical software version 27 and R software version 3.6.2 with a *p*-value < 0.05 considered statistically significant for a two-tailed test.

## Results

### Characteristics of the study population

The characteristics of the study population are shown in Table 1. Our study comprised 1,245,817 accumulated sleep records over 3 years from 1005 nights of sleep tracking among 7682 participants, 70.6% of which were weeknights. There were relatively even between seasons in this analysis across the 3 years, although the highest proportion of records came from autumn (27.8%). Over half of the study population resided in first-tier or super-first-tier cities and came from low-elevation regions. The mean age of the participants was 47.7 ± 13.8 years old. Individuals aged 18 to 64 years provided the most nights of the population. Males accounted for 74.1% of this analysis. The mean BMI of the study population was 24.4 ± 3.2 kg/m^2^, predominantly in the BMI normal group (43.5%).

**Table 1:** Characteristics of research data

Items	Number of participants
All participants	7682
Gender of participants, no. (%)	
Male	5689 (74.1%)
Female	1993 (25.9%)
Age of participants, mean (SD), years	47.7 (13.8)
Age groups of participants, no. (%)	
< 18 years	45 (0.6%)
18–44 years	4018 (52.3%)
45–64 years	2636 (34.3%)
≥ 65	983 (12.8%)
BMI of participants, mean (SD), kg/m^2^	24.4 (3.2)
BMI groups of participants, no. (%)	
Low weight (< 18.5)	243 (3.2%)
Normal (18.5–23.9)	3340 (43.5%)
Overweight (24–27.9)	2993 (39.0%)
Obesity (≥ 28)	1106 (14.4%)
City groups of participants, no. (%)	
Super first-tier cities	2426 (31.6%)
First-tier cities	1811 (23.6%)
Second-tier cities	1488 (19.4%)
Third-tier cities	960 (12.5%)
Fourth-tier cities	690 (9.0%)
Fifth-tier cities	307 (4.0%)
Altitude groups of participants, no. (%)	
≥ 1000 m	389 (5.1%)
< 1000 m	7293 (94.9%)
Smoking, no. (%)	
Never	5338 (69.5%)
Occasional	929 (12.1%)
Regular	345 (4.5%)
Daily	1070 (13.9%)
Drinking, no. (%)	
Never	2300 (29.9%)
Occasional	4419 (57.5%)
Regular	774 (10.1%)
Daily	189 (2.5%)
Total nights of sleep tracking, no.	1005
Accumulative sleep records, nights	1,245,817
Type of the night sleep records tracked in, no. (%)	
Weeknights	879,289 (70.6%)
Nights of rest day	366,528 (29.4%)
Seasons sleep records tracked in, no. (%)	
Spring (March to May)	326,332 (26.2%)
Summer (June to August)	284,066 (22.8%)
Autumn (September to November)	346,791 (27.8%)
Winter (December to February)	288,628 (23.2%)

*Abbreviations*: *IQR* Interquartile range, *SD* Standard deviation

**Fig. 2:**
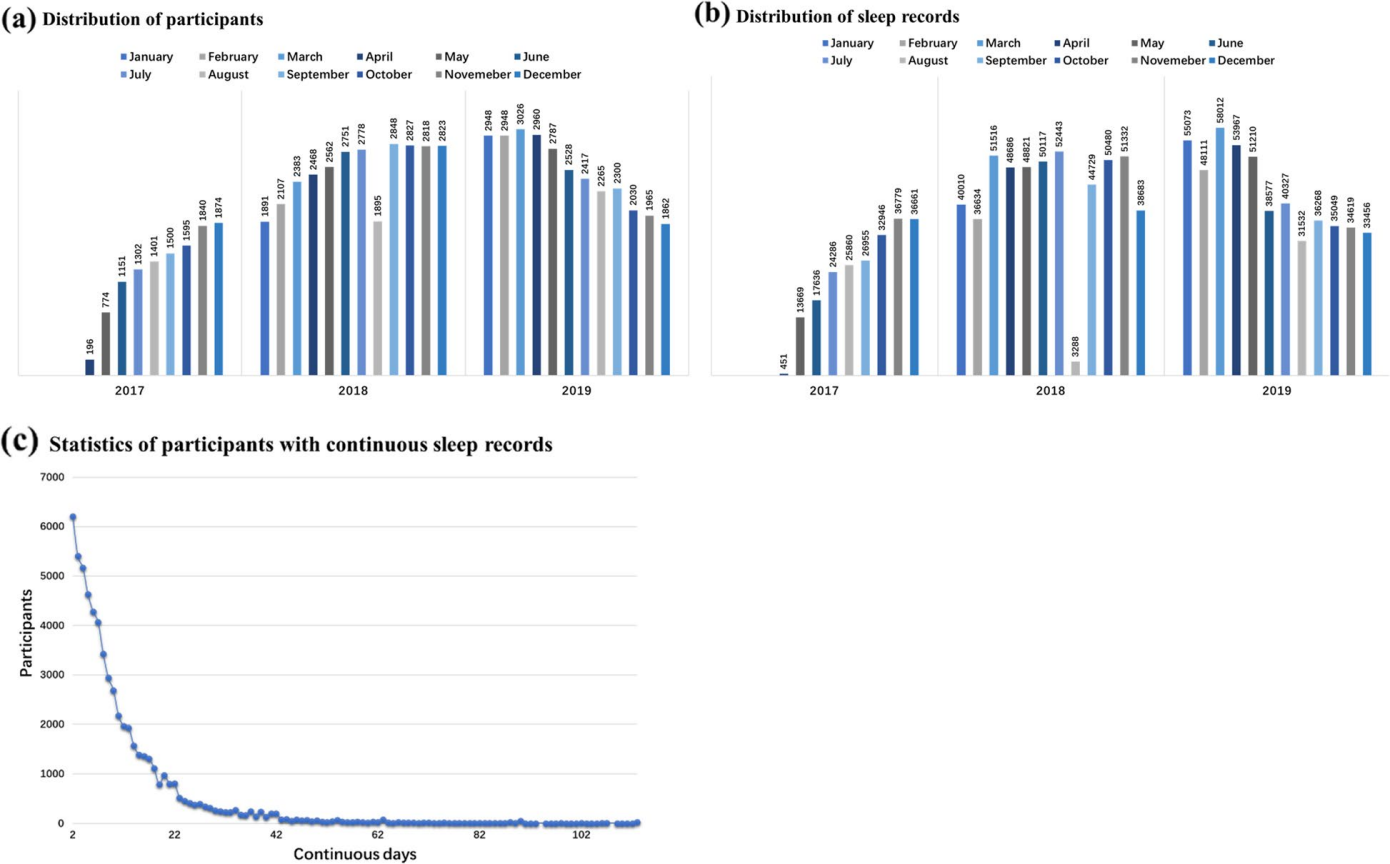
**a**–**c** Characteristics of data distribution. **a**, **b** The temporal distribution of participants and sleep records respectively. **c** The distribution of the number of participants in the continuous records of different lengths

Figure 2 a and b illustrate the temporal distribution of the overall data. The period of the data was from April 2017 to December 2019, with the largest amount derived from 2019. Specifically, the data volume peaked in March 2019, including 58,012 data from 3026 participants. Figure 2c shows the distribution of the number of participants in the continuous records of different lengths. The number of consecutive record days ranged from 2 to 113 days. In terms of the overall trend, the longer the consecutive days, the fewer the participants.

### Sleep parameters

The sleep parameters recorded by the bracelets are summarized in Table 2. Of the overall nights studied, the mean total sleep duration was 419.7 ± 87.3 min, ranging from 180 to 720 min. The deep sleep duration was 108.49 ± 49.77 min, and the light sleep duration was 311.20 ± 80.44 min, respectively. The average proportion of deep sleep in total sleep duration was 25.9% ± 10.9%, and the ratio of deep to light sleep duration was 0.38 ± 0.24. The average times of WASO per night of sleep were 0.90 ± 1.1. The duration of WASO was 9.27 ± 19.69 min, which accounted for 2.3% ± 5.09% of total sleep.

**Table 2:** Summary of parameters of the study population during the recording period

Sleep parameters	Mean (SD)	Median (IQR)	Min	Max
Total sleep duration, min	419.69 (87.29)	423 (366,476)	180	720
Deep sleep duration, min	108.49 (49.77)	104 (72,139)	8	505
Light sleep duration, min	311.20 (80.44)	311 (257,364)	37	680
Deep sleep duration/light sleep duration, %	0.38 (0.24)	0.34 (0.22,0.49)	0.01	9.22
Deep sleep duration/total sleep duration, %	25.94 (10.87)	25.14 (18.12, 32.87)	1.37	91.65
Times of WASO	0.90 (1.10)	1 (0, 1)	0	17
Times of WASO per hour of sleep	0.13 (0.16)	0.12 (0, 0.20)	0	3.31
Duration of WASO, min	9.27 (19.69)	0 (0, 7)	0	456
Duration of WASO/total sleep duration, %	2.28 (5.09)	0 (0, 1.73)	0	86.51

*Abbreviations*: *IQR* Interquartile range, *SD* Standard deviation, *WASO* Wake after sleep onset

### Ambient air pollutant concentrations

**Fig. 3:**
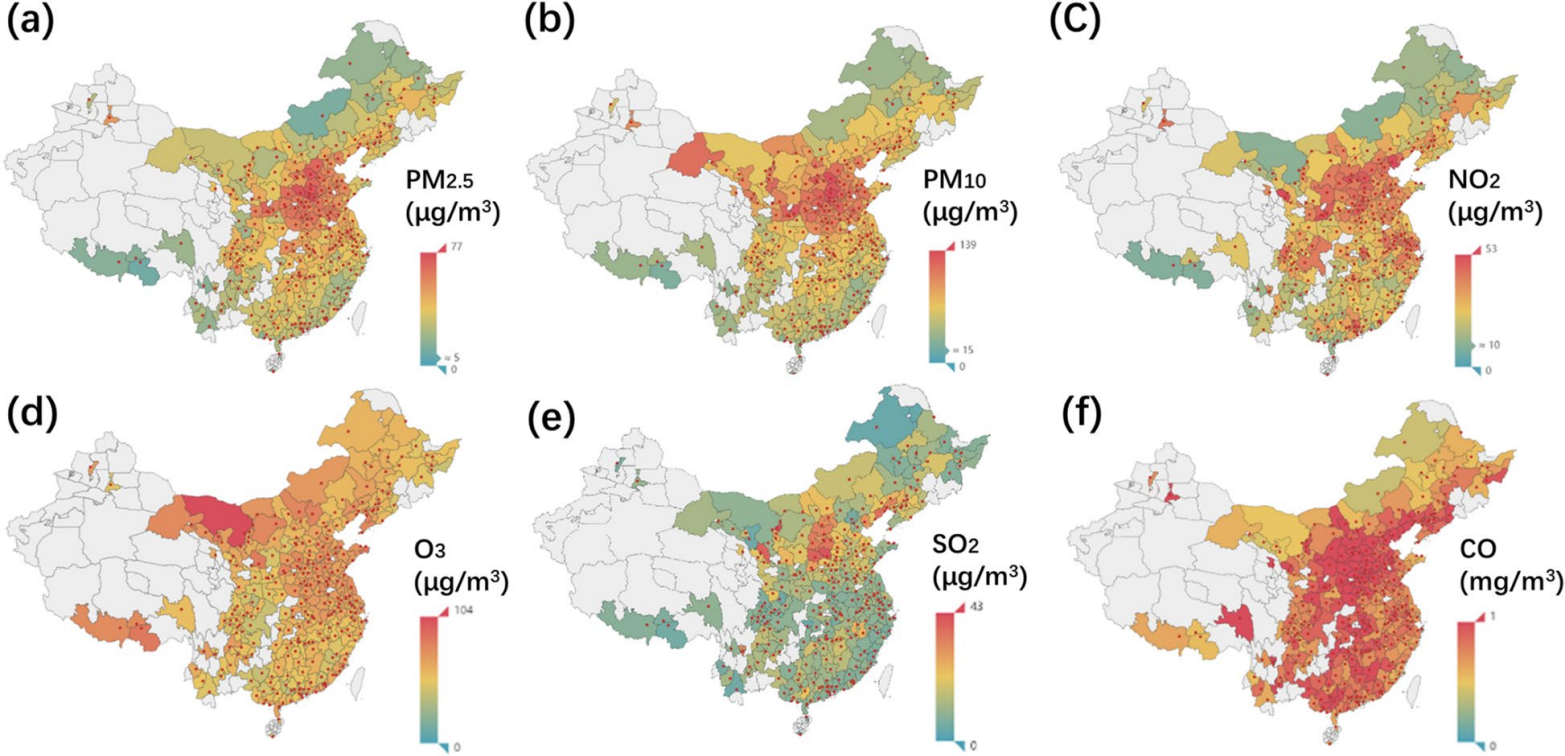
**a**–**f** Spatial distribution of long-term air pollutant concentration in participants’ residences. The red dots represent participants’ residential cities, and the color depth represents the concentration of each air pollutant. The World Health Organization air quality guidelines 2021 for PM_2.5_, PM_10_, NO_2_, O_3_, SO_2_, and CO concentrations were 5 μg/m^3^ (annual), 15 μg/m^3^ (annual), 10 μg/m^3^ (annual), 100 μg/m^3^(8-h average), 40 μg/m^3^ (24-h average), and 4 mg/m^3^ (24-h average), respectively

Table 3 shows the distribution of short- and long-term air pollutant levels in this study. The mean long-term concentrations of PM_2.5_, PM_10_, NO_2_, O_3_, SO_2_, and CO were 45.3 ± 13.7μg/m^3^, 77.5 ± 25.9μg/m^3^, 39.8 ± 9.0μg/m^3^, 61.0 ± 9.1μg/m^3^, 13.0 ± 7.7μg/m^3^, and 0.9 ± 0.2μg/m^3^ respectively. These values were higher than the WHO air quality guidelines [11]. Even the annual minimum of PM_2.5_ and PM_10_ exceeded the WHO standard (5 μg/m^3^ for PM_2.5_ and 15 μg/m^3^ for PM_10_). Figure 3 presents the spatial distribution of long-term air pollutant concentrations in participants’ residences. It was found that the participants mostly lived in developed regions where air pollution was severe, and the population density was high.

**Table 3:** Distribution of air pollutant concentrations in the study

	Short-term exposure							Long-term exposure
	Lag 0	Lag 0-1	Lag 0-2	Lag 0-3	Lag 0-4	Lag 0-5	Lag 0-6	
PM_2.5_								
Mean (SD)	42.4 (33.6)	42.3 (31.1)	42.3 (29.2)	42.3 (27.8)	42.2 (26.7)	42.2 (25.8)	42.2 (25.2)	45.3 (13.7)
Median (IQR)	33.0 (21.0, 52.0)	34.0 (22.5, 52.0)	34.7 (23.5, 52.0)	35.0 (24.3, 51.7)	35.4 (24.8, 51.6)	35.7 (25.3, 51.5)	36.0 (25.7, 51.3)	43.1 (36.3, 53.3)
Min	0	0	0	0	0	0	0	10.6
Max	771.0	613.0	463.0	370.0	327.8	302.0	280.0	115.3
PM_10_								
Mean (SD)	73.2 (53.0)	73.2 (48.8)	73.2 (46.0)	73.1 (44.0)	73.0 (42.5)	73.0 (41.3)	73.0 (40.4)	77.5 (25.9)
Median (IQR)	60.0 (40.0, 91.0)	61 (41.5, 90.5)	61.3 (42.7, 90.3)	62.0 (43.5, 90.0)	62.6 (44.2, 89.6)	63.0 (44.7, 89.8)	63.5 (45.1, 89.6)	73.3 (57.5, 88.7)
Min	0	0	0	0	0	0	0	25.1
Max	1908.0	1132.5	792.3	676.5	819.4	710.8	637.9	193.0
NO_2_								
Mean (SD)	38.4 (18.4)	38.4 (17.2)	38.4 (16.4)	38.4 (15.9)	38.3 (15.4)	38.3 (15.1)	38.3 (14.8)	39.8 (9.0)
Median (IQR)	35.0 (25.0, 49.0)	35.5 (26.0, 48.0)	36.0 (26.7, 47.7)	36.0 (27.0, 47.8)	36.2 (27.2, 47.7)	36.4 (27.3, 47.7)	36.4 (27.5, 47.6)	41.8 (34.8, 45.1)
Min	0	0	0	0	0	0	0	7.6
Max	181.0	161.0	158.3	147.0	141.6	136.2	130.1	63.0
O_3_								
Mean (SD)	60.3 (32.0)	60.2(30.4)	60.2(29.4)	60.2(28.7)	60.2(28.2)	60.3 (27.7)	60.3(27.4)	61.0 (9.1)
Median (IQR)	56.2(36.1, 80.4)	56.5(36.9, 79.5)	56.8 (37.5,79.3)	56.9 (38.0, 79.2)	57.3(38.3, 78.9)	57.4 (38.6,78.9)	57.6 (38.8, 78.7)	60.1(54.6, 67.7)
Min	1	1.9	2.5	2.8	2.8	2.8	3.1	33.2
Max	233.6	232.0	224.3	214.2	210.5	200.6	193.6	107.0
SO_2_								
Mean (SD)	11.2 (9.2)	11.2 (8.7)	11.2 (8.5)	11.2 (8.3)	11.2 (8.2)	11.2 (8.2)	11.2 (8.1)	13.0 (7.7)
Median (IQR)	9.0 (6.0, 13.0)	35.5 (26.0, 48.0)	9.0 (6.3, 13.0)	9.0 (6.5, 13.0)	9.0 (6.4, 13.0)	9.0 (6.5, 13.0)	9.0 (6.6, 13.0)	11.4 (8.0, 15.1)
Min	0	0	0	0	0	0	0	2.6
Max	342.0	161.0	225.7	215.0	207.8	202.8	192.1	115.4
CO								
Mean (SD)	0.8 (0.4)	0.8 (0.4)	0.8 (0.3)	0.8 (0.3)	0.8 (0.3)	0.8 (0.3)	0.8 (0.3)	0.9 (0.2)
Median (IQR)	0.8 (0.6, 1.0)	0.8 (0.6, 1.0)	0.8 (0.6, 1.0)	0.8 (0.6, 1.0)	0.8 (0.6, 1.0)	0.8 (0.6, 1.0)	0.8 (0.6, 1.0)	0.8 (0.7, 1.0)
Min	0	0	0	0	0	0	0	0.3
Max	6.4	5.6	5.7	5.4	4.8	4.7	4.3	2.4

*Abbreviations*: *CO* Carbon monoxide, *IQR* Interquartile range, *NO*_*2*_ Nitrogen dioxide, *O*_*3*_ Ozone, *PM*_*2.5*_ Particulate matter with aerodynamic diameter ≤ 2.5 μm, *PM*_*10*_ Particulate matter with aerodynamic diameter ≤ 10 μm, *SD* Standard deviation, *SO*_*2*_ Sulfur dioxide

The 7-day moving average levels of PM_2.5_, PM_10_, NO_2_, O_3_, SO_2_, and CO were 42.2 ± 25.2μg/m^3^, 73.0 ± 40.4μg/m^3^, 38.3 ± 14.8μg/m^3^, 60.3 ± 27.4μg/m^3^, 11.2 ± 8.1μg/m^3^, and 0.8 ± 0.3μg/m^3^ respectively. The trend for each pollutant varied in designative cumulative lag days. The moving averages of PM_2.5_, PM_10_, and NO_2_ gradually increased as the recording day approached (Lag0). The moving average of O_3_ concentration was lowest in Lag0-3 and had two peaks on Lag0 and Lag0-6, showing a U-shaped curve. The moving average of SO_2_ levels peaked at Lag0 and Lag0-3 and gradually decreased during the other periods. The moving averages of CO were essentially the same for cumulative lag days. In addition, a previous study [22] illustrated that the closer the cumulative days are to the record day, the larger the variations are for the values. Similar patterns were observed in our study.

### Overall data analysis

1. The long-term effects of ambient air pollutants on sleep parameters

**Fig. 4:**
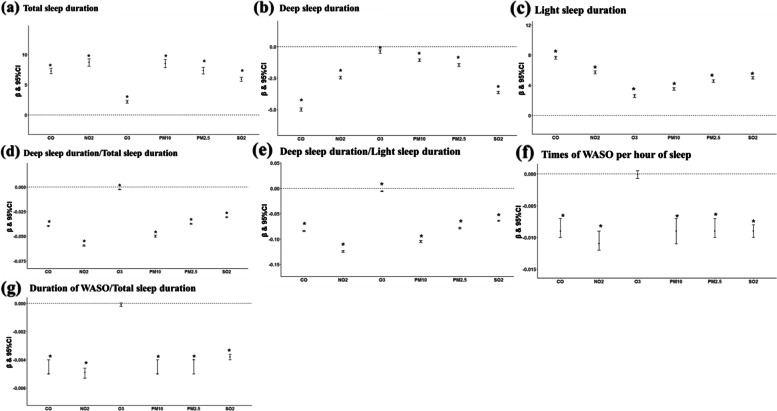
**a**–**g** Associations between sleep parameters and long-term exposures to ambient air pollutants. Data are *β* (95% CI). *β* indicates partial regression coefficient. Estimates were associated with per 1-interquartile range increase in concentration of each pollutant. Adjusted for age, sex, BMI, city development level, altitude, season, and the type of night. *p < 0.05. CI, confidence intervals; CO, carbon monoxide; NO_2_, nitrogen dioxide; O_3_, ozone; PM_2.5_, particulate matter with aerodynamic diameter ≤ 2.5 μm; PM_10_, particulate matter with aerodynamic diameter ≤ 10 μm; SO_2_, sulfur dioxide; WASO, wake after sleep onset

Figure 4 demonstrates the association between long-term exposure to air pollutants and sleep parameters. The adjusted mixed-effect models showed that a higher concentration of each air pollutant was associated with longer total sleep and light sleep durations, whereas with reduced deep sleep duration and proportion. Nitrogen dioxide had the greatest impact on the total sleep duration. Every 1-IQR increase in NO_2_ exposure prolonged the total sleep duration by 8.7 (8.08 to 9.32) minutes. Carbon monoxide was most closely related to both deep and light sleep duration, with each 1-IQR increase in CO shortening deep sleep duration by 5.0 (− 5.13 to − 4.89) minutes and prolonging light sleep duration by 7.7 (7.46 to 7.85) minutes. Statistically, elevated concentrations of each air pollutant significantly reduced the times of WASO per hour of sleep and the proportion of WASO duration, except for ozone, which was in contrast to a previous publication [16]. Although the regression coefficients were relatively low, they may still be meaningful because WASO rarely occurs during sleep.

2. The short-term effects of ambient air pollutants on sleep parameters

**Fig. 5:**
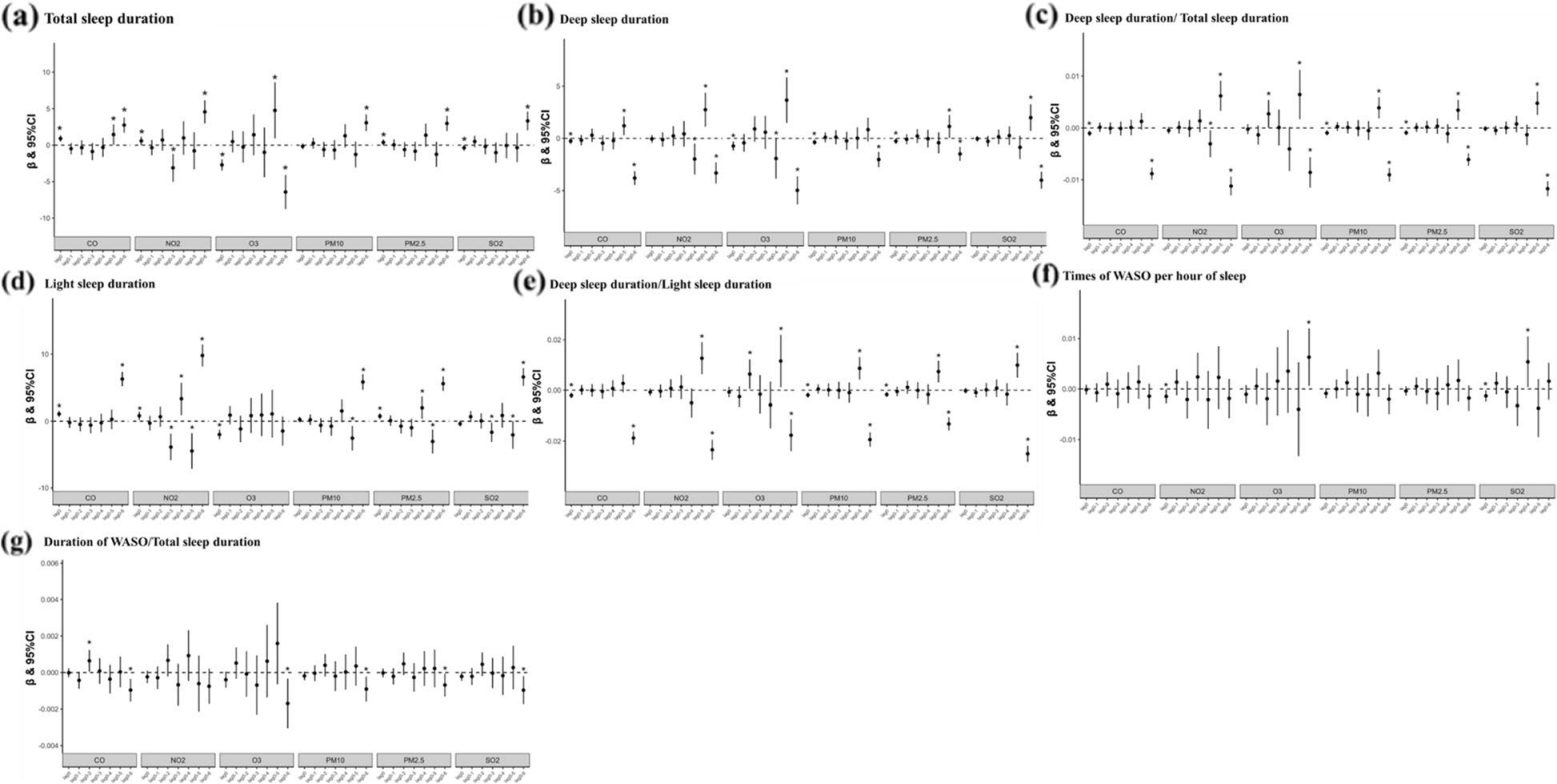
**a**–**g** Associations between sleep parameters and short-term exposure to ambient air pollutants. Data are *β* (95% CI). *β* indicates partial regression coefficient. Estimates were associated with per 1-interquartile range increase in concentration of each pollutant. Adjusted for age, sex, BMI, city development level, altitude, season, and the type of night. *p < 0.05. CI, confidence intervals; CO, carbon monoxide; NO_2_, nitrogen dioxide; O_3_, ozone; PM_2.5_, particulate matter with aerodynamic diameter ≤ 2.5 μm; PM_10_, particulate matter with aerodynamic diameter ≤ 10 μm; SO_2_, sulfur dioxide; WASO, wake after sleep onset

Figure 5 presents the associations between ambient air pollutant levels and sleep parameters on cumulative 0–6 days (from Lag0 to Lag0–6), adjusting for confounders. We observed that although the effect of the same air pollutant on sleep parameters on different cumulative days was inconsistent, the effect of each air pollutant on sleep parameters had a certain degree of similarity. Specifically, the majority of air pollutants had the greatest impact on sleep parameters at Lag0-6, including generally positive associations with total sleep and light sleep duration, and negative associations with both deep sleep and WASO, except for ozone, which had a negative association with total sleep duration and no significant association with light sleep duration. Conversely, some air pollutant (PM_2.5_, NO_2_, SO_2_, CO) levels were positively associated with deep sleep duration in Lag0-5, while their impacts were significantly smaller than those in Lag0-6. In summary, the cumulative effects from Lag0 to Lag0-5 were generally unset and insignificant. In contrast, the cumulative effects at Lag0-6 tended to become significant and comparable to the long-term effects but relatively less.

3. Subgroup analyses of the associations between long-term exposure to ambient air pollutants and sleep parameters

Since the short-term effects were unset and insignificant, we only estimated the association between long-term air pollution and sleep parameters classified according to age, sex, sleep duration, and season (Supplementary Table S2, S3, S4, S5).

First, the majority of ambient air pollutants in different subgroups still had a significant impact on sleep, which is generally consistent with the impact on the overall population. Second, most air pollutants’ effects on sleep parameters were significantly different between subgroups. When classified by sex (Supplementary Table S2), the associations were more apparent in the female group. In terms of age (Supplementary Table S3), the effects on total sleep, deep sleep, and light sleep durations were greater in the younger age group (age < 45 years), whereas the effect on WASO was more pronounced in older people (age ≥ 45 years). For those with longer sleep (≥ 7 h), the impacts on deep and light sleep durations were more remarkable. Nevertheless, the impact on deep sleep proportion (the ratio of deep sleep to total sleep and light sleep) was stronger in the shorter sleep duration group (< 7 h) (Supplementary Table S4). Considering the seasons (Supplementary Table S5), the effects of air pollutants on total sleep and light sleep durations were more significant in the cold seasons. The effects on WASO were more pronounced in the warm season. Finally, an interesting phenomenon is that the effects of ozone on some sleep parameters in the subgroups were inconsistent with the overall effects. The significance of its effect in different subpopulations showed partly opposite trends to those of other pollutants; for instance, ozone exposure prolonged deep sleep duration in females, younger individuals, and those in cold seasons. Additionally, the effect was greater in males.

### Stratified analyses

We conducted stratified analyses, as described above, to reduce repeated measures of outcomes and exposures meanwhile accounting for individual variation.

**Fig. 6:**
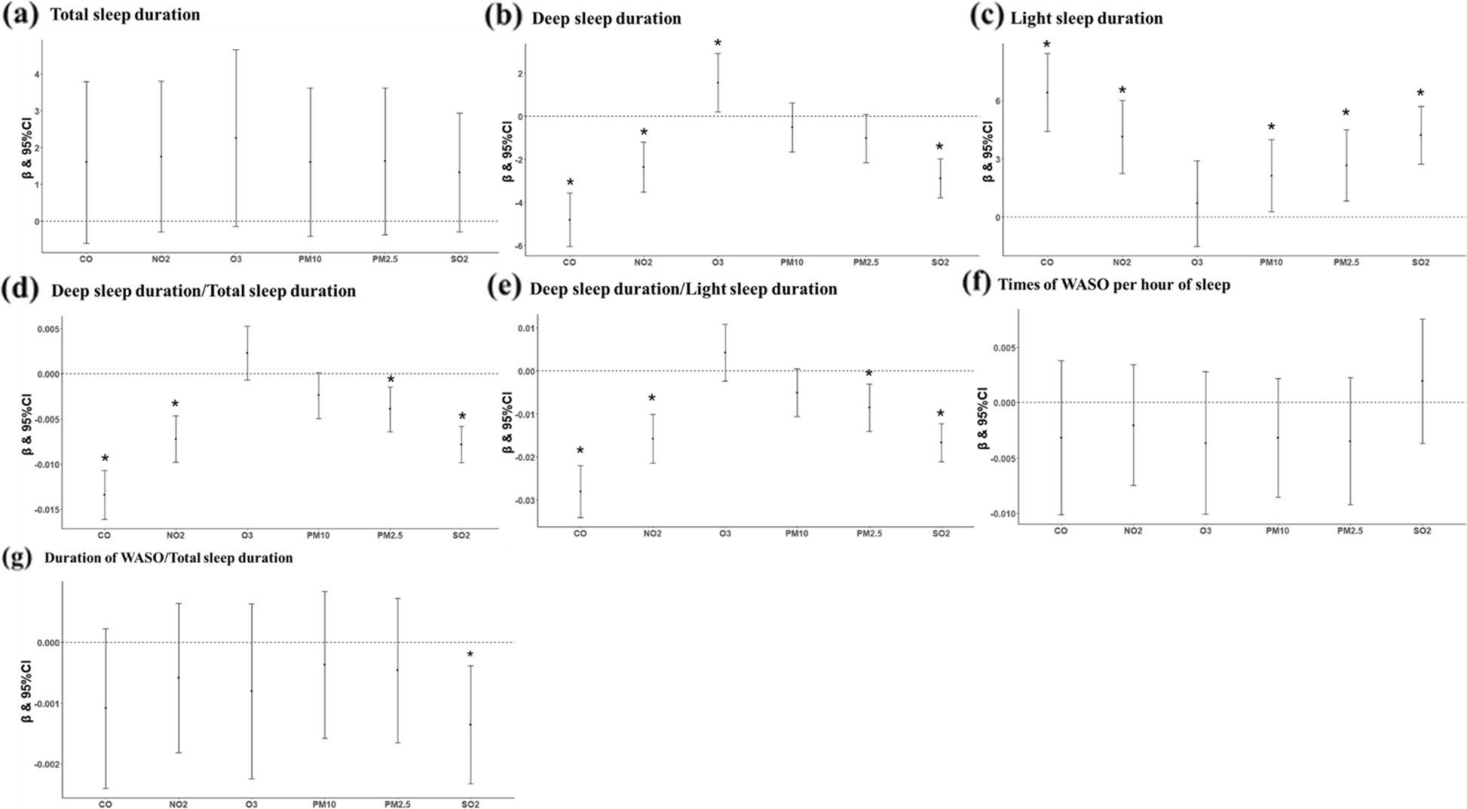
**a**–**g** Stratified analysis 1—Effect of long-term pollutant exposure on sleep parameters. Use the method of extracting records every 365 days intervals. Data are *β* (95% CI). *β* indicates partial regression coefficient. Estimates were associated with per 1-interquartile range increase in concentration of each pollutant. Adjusted for age, sex, BMI, city development level, altitude, season, and the type of night. *p < 0.05. CI, confidence intervals; CO, carbon monoxide; NO_2_, nitrogen dioxide; O_3_, ozone; PM_2.5_, particulate matter with aerodynamic diameter ≤ 2.5μm; PM_10_, particulate matter with aerodynamic diameter ≤ 10 μm; SO_2_, sulfur dioxide; WASO, wake after sleep onset

First, we used the method for sampling at time intervals of 7 days and 365 days. After data screening, 11,413 records remained for the analysis of long-term effects, and 181,392 records were for the analysis of short-term effects. The results were generally similar to the overall results (Fig. 6, Supplementary Figure S1). The long-term effects of CO on deep and light sleep duration remained strongest, with each 1-IQR increase in CO shortening 4.8 (− 6.05 to − 3.59) minutes of deep sleep and prolonging 6.4 (4.42 to 8.41) minutes of light sleep. Although the effects of individual air pollutants on some sleep parameters lost statistical significance, they remained consistent with the trends in the overall results. For instance, all pollutants had the trend of prolonging the total sleep duration and shortening the duration and proportion of WASO.

**Fig. 7:**
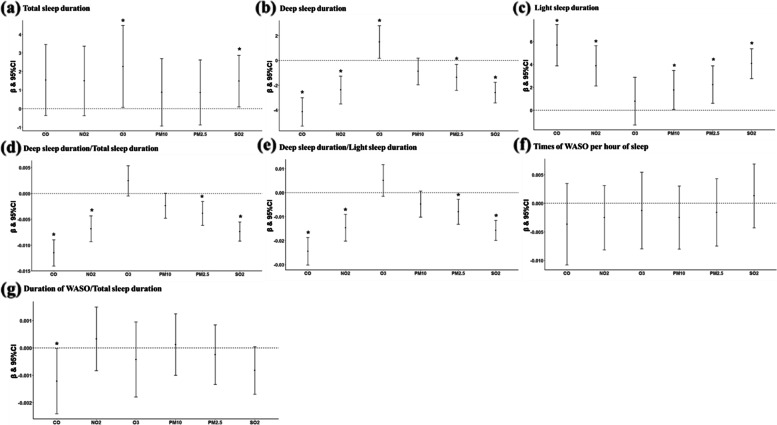
**a**–**g** Stratified analysis 2—Effect of long-term pollutant exposure on sleep parameters. Use the method of calculating the average value of the first data set of each subject. Data are *β* (95% CI). *β* indicates partial regression coefficient. Estimates were associated with per 1-interquartile range increase in concentration of each pollutant. Adjusted for age, sex, BMI, city development level, altitude, season, and the type of night. *p < 0.05. CI, confidence intervals; CO, carbon monoxide; NO_2_, nitrogen dioxide; O_3_, ozone; PM_2.5_, particulate matter with aerodynamic diameter ≤ 2.5μm; PM_10_, particulate matter with aerodynamic diameter ≤ 10 μm; SO_2_, sulfur dioxide; WASO, wake after sleep onset

Second, we adopted the method for calculating the averages of the outcome parameters. In total, 7682 and 114,194 records were utilized for long- and short-term effect analyses. The results were also consistent with the overall results, even though the associations were relatively fewer (Fig. 7, Supplementary Figure S2). For example, each 1-IQR increase in CO exposure was associated with 4.1 (− 5.28 to − 3.01) minutes shorter deep sleep and 5.7 (3.88 to 7.48) minutes longer light sleep. In summary, stratified analyses proved the robustness of the overall results from multiple perspectives.

## Discussion

In this real-world big data analysis of sleep records from consumer wearable devices in the Chinese population, greater exposure to both long- and short-term ambient air pollution was associated with longer total sleep duration and reduction in deep sleep and awake time during sleep.

To our knowledge, this is the first study to illuminate the associations between ambient air pollution and sleep characteristics through big data analysis of users of a popular sleep tracker on this platform for 3 years and over 1 million nights. Additionally, this study not only focused on the effects of long-term air pollution exposure but also examined the relationship between short-term air pollution exposure and sleep outcomes, which remains limited in the current literature. Furthermore, this study is one of the few studies to evaluate the general effect of ambient air pollution on sleep, including inhalable particulate matter (PM_2.5_, PM_10_), nitrogen dioxide, sulfur dioxide, carbon monoxide, and ozone.

Recently, an increasing number of studies have focused on the effects of air pollution exposure on sleep health, demonstrating an overall adverse effect of various air pollutants on sleep across the lifespan [23]. However, these findings largely depend on self-report questionnaires [12–14, 24–26], which have been proven to vary widely from objective sleep trackers. As an emerging technical device, bracelets are portable, commercially available, and feasible to detect sleep and have therefore become increasingly popular among the general population in recent years, presenting researchers with an opportunity to analyze the big data captured by these devices and explore the effects of long-term and short-term air pollution exposure on population sleep health.

### Long-term exposures and sleep characteristics

In our study, long-term exposure to each air pollutant was positively associated with total sleep duration and negatively associated with deep sleep duration. Ours is the first large-scale study to demonstrate the association of deep sleep with ambient air pollution exposure, revealing that although people tend to have prolonged total sleep duration with increasing air pollutant concentrations, their sleep quality might remain poor due to the reduction of deep sleep. Deep sleep is a homeostatic process that reflects the restorative role of sleep [27]. Increasing evidence supports the crucial role of deep sleep in modulating a multitude of physiological processes, including memory consolidation [28], energy conservation [29], clearance of metabolites [30], and immunity [31]. The relationship between air pollutant concentration and total sleep duration remains controversial. Our result is in line with findings from a small prospective cohort study in the USA that recruited 98 participants with previous a diagnosis of episodic migraines and demonstrated that greater ozone exposure resulted in approximately 7 min longer sleep duration at night [32]. However, the study population was not representative, and the duration of air pollution exposure was insufficient. Contrarily, other studies have demonstrated negative associations between higher long-term air pollutant levels and sleep duration, but in specific subpopulations such as female teachers [33], preschoolers [34], and college freshmen [13], most of which were assessed by self-reported questionnaires. Furthermore, several studies [35–40] examined the specific effects of ambient air pollutant exposure on sleep-disordered breathing (SDB), which is generally measured by the apnea-hypopnea index (AHI) and oxygen desaturation index (ODI). They reported a positive association between SDB and air pollution. A significant deficit in deep sleep has also been observed in patients with SDB [41]. These studies might suggest a mechanism for the negative association between air pollutants and deep sleep duration.

Another novel point of our study lies in the investigation of the associations between both arousal time and arousal frequency and ambient air pollution, showing that elevated concentrations of air pollutants reduced times of WASO (wake after sleep onset) and duration of WASO, whereas ozone had no significant effect on WASO. A limited number of previous studies have examined the association between air pollution and WASO, but in specific populations with small sample scales and shorter observation times. A study of 98 participants with episodic migraine exploring the association between air pollution exposure and WASO over an average of 45 days reported modest positive associations between ozone and WASO. In contrast, lower SO_2_ and CO were associated with high WASO [32]. Another study, contrary to our conclusion, reported that PM_2.5_ levels in metal fumes were positively associated with wake times during sleep, as measured by actigraphy, among 16 welding workers in China [16]. Our results are based on big-data analysis of long-term exposure duration. Therefore, we hypothesized that even if elevated concentrations of ambient air pollutants increase total sleep and reduce WASO, the proportion of deep sleep decreases, thus leading to low sleep efficiency and poor sleep quality. This hypothesis might be confirmed from the other aspect. In 39,259 Chinese rural residents, poor sleep quality, evaluated by the Pittsburgh Sleep Quality Index (PSQI), was associated with an increase in long-term exposure to PM_2.5_, PM_10_, and NO_2_ [12]. In 59,574 children from northeastern China, sleep disorders were associated with increased pollutants [26].

### Short-term exposures and sleep characteristics

In the present study, the effects of short-term exposure on sleep characteristics were also investigated. We found that the effects of short-term exposure to all air pollutants were most pronounced at Lag0-6 and partially resemble the long-term effects, including longer total sleep and light sleep duration, shorter deep sleep duration, and WASO. A study from China, including 12,000 freshmen, observed a positive association between weekly PM_2.5_ exposure and sleep duration in self-reported questionnaires [14], which was in line with our study. From Lag0 to Lag0-5, their impacts were somewhat unset and insignificant. To the best of our knowledge, this is the first study to observe associations between multiple short-term air pollutant exposures and sleep parameters, suggesting that we ought to emphasize the negative impact of short-term air pollutant exposure on sleep in the meantime, as merely a 1-week exposure has the potential to evolve toward a similar long-term exposure.

The majority of epidemiological studies have explored the association between long-term exposure and sleep; however, few studies have highlighted the association between short-term exposure and sleep. Therefore, this study is also novel because we not only evaluated the effects of both long- and short-term exposures but also inquired into their intrinsic relationship. In 4312 adults from Northern Taiwan urban areas, Shen and colleagues [35] examined the associations between daily, weekly mean, and annual PM_2.5_ exposure and SDB. The study found that both long- and short-term exposure increases in PM_2.5_ levels were associated with SDB, and the effect of long-term PM_2.5_ exposure was more significant. In other studies examining the effects of long- and short-term air pollution exposure on blood pressure [42], cardiovascular diseases [43], and psychiatric disorders [44], consistency between long- and short-term effects was also observed to some extent among which was more pronounced in the long-term. The stronger effect of long-term exposure can be explained by the cumulative damage of short-term exposure.

### Subgroup analyses of long-term effects

The results of subgroup analyses are similar to the analysis results of the total population, which further proves the credibility of our conclusion that the influence of ambient air pollution on sleep is consistent in different seasons and populations with different genders, ages, and sleep durations.

Furthermore, when classified by sex, the relationship between long-term exposure to air pollution and sleep was generally greater in females, which is in line with previous studies [45–47]. On the one hand, long-term exposure to ambient air pollution was identified as a risk factor for mental disorders, such as depression [48]. Sleep disorder is frequently regarded as a symptom of a sub-health psychological state. Correspondingly, women are relatively more emotional and, thus, more susceptible to the effects of air pollution on sleep. On the other hand, a recent study reported that compared to male patients with OSA, stronger effects of air pollution on SDB parameters were observed in female patients [46]. This evidence not only supports our results but more significantly suggests that we should pay particular attention to the impact of air pollution on women’s sleep.

Age effect has also been investigated in our study. More significant associations between long-term air pollutant exposure and sleep parameters were observed in the younger population. Previous Chinese studies focusing on the association between long-term exposure to air pollution and sleep quality [12] or diabetes [9] also reported stronger effects in the younger subgroup. One plausible explanation for this discrepancy might be the activity pattern. Young people have more work and entertainment activities and are exposed to more air pollutants while aging individuals are less exposed to ambient air pollutants due to physical limitations. Nevertheless, the effect of air pollution on WASO is more pronounced in older adults. Increasing involuntary awakening during sleep is one of the hallmarks of human sleep alterations with age. Thus, fragile regulation of sleep/wakefulness and sleep homeostasis in older people might be more vulnerable to air pollution [49].

Another innovation of our study lies in stratification according to sleep duration. This is the first study to focus on the impact of air pollution on populations with different sleep durations. Both short (< 7 h) and long sleep durations (> 9 h) seem to be detrimental to health. Because few people in our study slept for more than 9 h, we classified sleep duration by 7 h as the threshold. Subgroup analyses showed that the impact of air pollution on sleep was greater in those who slept for more than 7 h. Participants who sleep shorter than 7 h might frequently suffer from other factors that more significantly affect sleep duration, such as various activities, working pressure, insomnia, and other mental and somatic disorders, thus obscuring the impact of air pollution on sleep. But the impact on deep sleep proportion was stronger in the shorter sleep duration group, which might be due to that shorter sleep durations influenced the results of such ratios rather than a direct effect of air pollutants.

Of note, the effects of ozone on sleep indicators are not entirely consistent with the overall effect, and the significance of its effect in different subgroups has a partially opposite trend to that of other air pollutants. Previous studies investigating the effects of air pollution on human health, such as arterial pressure [50], blood lipids [51], and circulating inflammatory markers [52], similarly found discrepant characteristics in the effects of ozone. However, to our knowledge, there is limited data to explain this phenomenon. We inferred that these complicated associations are affected by distinct biological mechanisms of diverse air pollutants [23]. In addition, the negative correlation between ozone concentration and other air pollutants might also play a role. Therefore, further studies are warranted.

### Strengths and limitations

The strengths of our analysis include the big data used to perform the analyses of sleep data from a large and representative population over 3 years. The effects of multiple common ambient air pollutants on sleep were comprehensively studied, and several factors strongly influencing sleep were controlled. We also investigated the effects of short- and long-term air pollution exposure in the same sample compared their similarities and differences and analyzed their intrinsic associations. Moreover, the association between deep sleep and air pollutant exposure was innovatively highlighted. Last but not least, we conducted multifaceted subgroup analyses to demonstrate the credibility of our results and compare discrepancies in the effects of air pollutants on sleep according to population characteristics, sleep duration, and seasonal conditions.

Several limitations should be acknowledged. First, although wearable sleep-tracking devices have been proven to be reasonably sensitive and can identify the sleep cycle with a certain degree of accuracy [53, 54], some recent studies have reported that they tend to underestimate sleep disruptions and overestimate total sleep times compared to polysomnography (PSG) [54, 55]. Second, we did not test the accuracy of this sleep-tracking device by using PSG or medical actigraphy. Third, because of the intermittent wearing of bracelets in the real world and the privacy policies of producers, we cannot continuously and regularly analyze the sleep of individuals for a sufficient duration. As a consequence, all sleep data were studied in units per night rather than conventionally per person. However, big data analysis and the mixed-effects model incorporate overall data, which can reduce confounding factors to some extent. The corresponding sleep research applied a similar analysis method [56]. Fourth, exposure levels were assigned using data from the nearest air monitor rather than personal air pollution exposure data, which may have misclassified some participants by randomly underestimating exposure in some and over-estimating exposure in others, and also overlooked the indoor air pollutant exposures. Fifth, although we adjusted for several confounders, there is still a possibility that unmeasured factors, such as temperature, humidity, traffic noise, and light, partly contributed to the associations. It should be clarified that we have obtained temperature and humidity data from multiple air monitoring stations across the country. However, because our study covered a wide geographic range of provinces and cities in China, the temperature and humidity data from decentralized monitoring stations cannot accurately reflect the actual exposure of the participants. Therefore, we did not adopt the temperature and humidity data in the study but took the season as a covariate, which is closely related to both temperature and humidity. Sixth, we did not consider multi-pollutant models because of strong correlations between the studied air pollutants. Seventh, we could not collect all the information of participants due to the limitations of the device app. Important information on comorbidities is missing. It cannot be excluded that the findings are related to subjects with diseases and maybe healthy persons show no alterations. Finally, repeated measures of outcomes and exposures might lead to potential bias, we thus supplemented the stratified analyses to demonstrate the robustness of the overall results.

## Conclusions

We analyzed sleep data from over 1 million nights captured by a consumer wearable sleep-tracking device over 3 years in the Chinese population. Our findings show that both short- and long-term exposure to ambient air pollution is associated with sleep characteristics, among which the cumulative effects of 1-week exposure tended to be comparable to those of long-term exposure. Generally, although people tend to have prolonged total sleep duration with increasing air pollutant concentrations, their sleep quality may remain poor due to the reduction of deep sleep. Subgroup analyses indicated greater effects on the individuals who were female, younger (< 45 years), slept longer (≥ 7 h), and in cold seasons, but the pattern of effects was mixed. More evidence should confirm these associations and clarify the biological mechanisms. In addition, researchers and sleep-tracking developers could collaborate on more stable sleep-tracking and accurate algorithms to facilitate large-scale studies for objective sleep evaluation.

## Supplementary Information

**Additional file 1: Supplementary Table S1.** Pearson’s correlation coefficients for long-term exposures to ambient air pollutants. **Supplementary Table S2.** Adjusted subgroup analysis of the associations between sleep parameters and long-term exposures to ambient air pollutants by sex. **Supplementary Table S3.** Adjusted subgroup analysis of the associations between sleep parameters and long-term exposures to ambient air pollutants by age. **Supplementary Table S4.** Adjusted subgroup analysis of the associations between sleep parameters and long-term exposures to ambient air pollutants by sleep duration. **Supplementary Table S5.** Adjusted subgroup analysis of the associations between sleep parameters and long-term exposures to ambient air pollutants by season. **Supplementary Table S6.** Associations between sleep parameters and long-term exposures to ambient air pollutants when adjusting for year, month, and day of week. **Supplementary Table S7.** Associations between sleep parameters and short-term exposure to ambient air pollutants when adjusting for year, month, and day of week. **Supplementary Figure S1.** (a-g) Stratified analysis 1—Effect of short-term pollutant exposure on sleep parameters. **Supplementary Figure S2.** (a-g) Stratified analysis 2—Effect of short-term pollutant exposure on sleep parameters.

## Data Availability

Data from this study are available from the corresponding authors upon reasonable request. Raw data on the main pollutants are available on the open website of the urban air quality of the Ministry of Ecology and Environment of the People’s Republic of China.
