# Supplementary material for: The long-term and short-term effects of ambient air pollutants on sleep characteristics in the Chinese population: big data analysis from real world by sleep records of consumer wearable devices
Source: BMC Med. 2023 Mar 8;21:83. doi: 10.1186/s12916-023-02801-1 (PMC9993685; doi:10.1186/s12916-023-02801-1)
Supplement: Supplementary file 1 — Additional file 1: Supplementary Table S1. Pearson’s correlation coefficients for long-term exposures to ambient air pollutants. Supplementary Table S2. Adjusted subgroup analysis of the associations between sleep parameters and long-term exposures to ambient air pollutants by sex. Supplementary Table S3. Adjusted subgroup analysis of the associations between sleep parameters and long-term exposures to ambient air pollutants by age. Supplementary Table S4. Adjusted subgroup analysis of the associations between sleep parameters and long-term exposures to ambient air pollutants by sleep duration. Supplementary Table S5. Adjusted subgroup analysis of the associations between sleep parameters and long-term exposures to ambient air pollutants by season. Supplementary Table S6. Associations between sleep parameters and long-term exposures to ambient air pollutants when adjusting for year, month, and day of week. Supplementary Table S7. Associations between sleep parameters and short-term exposure to ambient air pollutants when adjusting for year, month, and day of week. Supplementary Figure S1. (a-g) Stratified analysis 1—Effect of short-term pollutant exposure on sleep parameters. Supplementary Figure S2. (a-g) Stratified analysis 2—Effect of short-term pollutant exposure on sleep parameters. [file 12916_2023_2801_MOESM1_ESM.docx]

**Supplemental Materials**

The long-term and short-term effects of ambient air pollutants on sleep characteristics in the Chinese population:

big data analysis from real-world by sleep records of consumer wearable devices

Peining Zhou, Jing Ma*, Xueying Li, Yixue Zhao, Kunyao Yu, Rui Su, Rui Zhou, Hui Wang, Guangfa Wang*

**Supplementary Table S1:** **Pearson's correlation coefficients for long-term exposures to ambient air pollutants**

|  | Spearman correlation coefficients | | | | | |
| --- | --- | --- | --- | --- | --- | --- |
|  | PM_2.5_ | PM_10_ | NO_2_ | O_3_ | SO_2_ | CO |
| PM_2.5_ | 1.000 | 0.916* | 0.624* | -0.008* | 0.408* | 0.693* |
| PM_10_ |  | 1.000 | 0.533* | 0.015* | 0.522* | 0.724* |
| NO_2_ |  |  | 1.000 | -0.105* | 0.178* | 0.464* |
| O_3_ |  |  |  | 1.000 | 0.093* | -0.196* |
| SO_2_ |  |  |  |  | 1.000 | 0.620* |
| CO |  |  |  |  |  | 1.000 |

Abbreviations: CO, carbon monoxide; NO_2_, nitrogen dioxide; O_3_, ozone; PM_2.5_, particulate matter with aerodynamic diameter ≤2.5 μm; PM_10_, particulate matter with aerodynamic diameter ≤10 μm; SO_2_, sulfur dioxide.

*Statistically significant correlation (p < 0.05).

**Supplementary Table S2: Adjusted subgroup analysis of the associations between sleep parameters and long-term exposures to ambient air pollutants by sex**

|  | **Male** | | **Female** | | **P value for interaction** |
| --- | --- | --- | --- | --- | --- |
|  | β（95% CI） | P value | β（95% CI） | P value |  |
| Total sleep duration，min | | | | | |
| PM_2.5_ | 2.958(2.736 to 3.179) | 0.000 | 3.548 (3.147 to 3.949) | 0.000 | 0.010 |
| PM_10_ | 2.284 (2.066 to 2.500) | 0.000 | 3.007 (2.618 to 3.396) | 0.000 | 0.001 |
| NO_2_ | 3.473 (3.256 to 3.690) | 0.000 | 2.420 (2.041 to 2.798) | 0.000 | 0.000 |
| O_3_ | 2.209 (1.955 to 2.463) | 0.000 | 2.075 (1.612 to 2.537) | 0.000 | 0.613 |
| SO_2_ | 1.160 (0.986 to 1.335) | 0.000 | 1.930 (1.628 to 2.233) | 0.000 | 0.000 |
| CO | 2.265 (2.026 to 2.504) | 0.000 | 3.961 (3.536 to 4.386) | 0.000 | 0.000 |
| Deep sleep duration，min | | | | | |
| PM_2.5_ | -0.846(-0.972 to -0.721) | 0.000 | -3.581 (-3.807 to -3.354) | 0.000 | 0.000 |
| PM_10_ | -0.490 (-0.613 to -0.368) | 0.000 | -3.065 (-3.284 to -2.845) | 0.000 | 0.000 |
| NO_2_ | -1.778 (-1.900 to -1.655) | 0.000 | -4.890 (-5.103 to -4.676) | 0.000 | 0.000 |
| O_3_ | -0.591 (-0.734 to -0.448) | 0.000 | 0.326 (0.065 to 0.587) | 0.015 | 0.000 |
| SO_2_ | -3.381 ( -3.479 to -3.283) | 0.000 | -4.587 (-4.757 to -4.416) | 0.000 | 0.000 |
| CO | -4.470 (-4.605 to -4.335) | 0.000 | -6.872 (-7.111 to -6.632) | 0.000 | 0.000 |
| Deep sleep duration/Total sleep duration | | | | | |
| PM_2.5_ | -0.004 (-0.004 to -0.004) | 0.000 | -0.011 (-0.011 to -0.010) | 0.000 | 0.000 |
| PM_10_ | -0.003(-0.003 to -0.002) | 0.000 | -0.009 (-0.010 to -0.009) | 0.000 | 0.000 |
| NO_2_ | -0.007 (-0.007 to -0.006) | 0.000 | -0.013(-0.014 to -0.013) | 0.000 | 0.000 |
| O_3_ | -0.003 (-0.003 to -0.003) | 0.000 | -0.001(-0.002 to -0.000) | 0.000 | 0.000 |
| SO_2_ | -0.009(-0.009 to -0.009) | 0.000 | -0.012(-0.013 to -0.012) | 0.000 | 0.000 |
| CO | -0.013(-0.013 to -0.012) | 0.000 | -0.019 (-0.020 to -0.019) | 0.000 | 0.000 |
| Light sleep duration，min | | | | | |
| PM_2.5_ | 3.804 (3.600 to 4.009) | 0.000 | 7.129 (6.759 to 7.4989) | 0.000 | 0.000 |
| PM_10_ | 2.774 (2.573 to 2.974) | 0.000 | 6.072 (5.713 to 6.431) | 0.000 | 0.000 |
| NO_2_ | 5.251 (5.051 to 5.451) | 0.000 | 7.309(6.961 to 7.658) | 0.000 | 0.000 |
| O_3_ | 2.800 (2.566 to 3.034) | 0.000 | 1.749(1.322 to 2.176) | 0.000 | 0.000 |
| SO_2_ | 4.542 (4.381 to 4.702) | 0.000 | 6.517 (6.238 to 6.796) | 0.000 | 0.000 |
| CO | 6.735 (6.515 to 6.955) | 0.000 | 10.832 (10.441 to 11.224) | 0.000 | 0.000 |
| Deep sleep duration/ Light sleep duration | | | | | |
| PM_2.5_ | -0.008 (-0.009 to -0.008) | 0.000 | -0.026 (-0.028 to -0.025) | 0.000 | 0.000 |
| PM_10_ | -0.006(-0.006 to -0.005) | 0.000 | -0.023 (-0.024 to -0.022) | 0.000 | 0.000 |
| NO_2_ | -0.014 (-0.014 to -0.013) | 0.000 | -0.031(-0.032 to -0.030) | 0.000 | 0.000 |
| O_3_ | -0.006(-0.007 to -0.006) | 0.000 | -0.004(-0.005 to -0.003) | 0.000 | 0.002 |
| SO_2_ | -0.019(-0.019 to -0.018) | 0.000 | -0.028(-0.029 to -0.027) | 0.000 | 0.000 |
| CO | -0.025(-0.026 to -0.025) | 0.000 | -0.045(-0.046 to -0.044) | 0.000 | 0.000 |
| Times of WASO per hour of sleep | | | | | |
| PM_2.5_ | -0.005 (-0.005 to -0.004) | 0.000 | -0.008(-0.009 to -0.007) | 0.000 | 0.000 |
| PM_10_ | -0.004 (-0.004 to -0.003) | 0.000 | -0.007(-0.007 to -0.006) | 0.000 | 0.000 |
| NO_2_ | -0.004(-0.004 to -0.003) | 0.000 | -0.007(-0.008 to -0.006) | 0.000 | 0.000 |
| O_3_ | 2.29E-04(-4.20E-04 to 8.81E-04) | 0.491 | -0.001(-0.002 to 4.33E-05) | 0.059 | 0.045 |
| SO_2_ | -0.002(-0.003 to -0.001) | 0.000 | 3.42E-04(-0.001 to 0.001) | 0.468 | 0.000 |
| CO | -0.005(-0.006 to -0.005) | 0.000 | -0.006(-0.007 to -0.005) | 0.000 | 0.333 |
| Duration of WASO/ Total sleep duration | | | | | |
| PM_2.5_ | -0.001(-0.001 to -0.001) | 0.000 | -0.001(-0.001 to -0.001) | 0.000 | 0.043 |
| PM_10_ | -0.001(-0.001 to -0.001) | 0.000 | -0.001 (-0.001 to -0.001) | 0.000 | 0.027 |
| NO_2_ | -0.001 (-0.001 to -4.5E-04) | 0.000 | -0.001(-0.001 to -0.001) | 0.000 | 0.001 |
| O_3_ | -3.7E-04(-0.001 to -2.2E-04) | 0.000 | 8.85E-04(6.15E-04 to 0.001) | 0.003 | 0.000 |
| SO_2_ | -0.001 (-0.001 to -0.001) | 0.000 | -0.001 (-0.002 to -0.001) | 0.000 | 0.001 |
| CO | -0.002 (-0.002 to -0.001) | 0.000 | -0.002(-0.003 to -0.002) | 0.000 | 0.000 |

Note: data are β (95%CI). β indicates partial regression coefficient. Estimates were associated with per 1-interquartile range increase in concentration of each pollutant (PM_2.5_, 17.0µg/m^3^; PM_10_, 31.2µg/m^3^; NO_2_, 10.3µg/m^3^; O_3_ 13.1µg/m^3^; SO_2_ 7.1µg/m^3^; CO 0.3 mg/m^3^). Adjusted for age, sex, BMI, city development level, altitude, season, and the type of nights.

Abbreviations: CI, confidence intervals; CO, carbon monoxide; NO_2_, nitrogen dioxide; O_3_, ozone; PM_2.5_, particulate matter with aerodynamic diameter ≤2.5 μm; PM_10_, particulate matter with aerodynamic diameter ≤10 μm; SO_2_, sulfur dioxide; WASO: wake after sleep onset.

**Supplementary Table S3: Adjusted subgroup analysis of the associations between sleep parameters and long-term exposures to ambient air pollutants by age**

|  | **Age < 45 years** | | **Age ≥ 45 years** | | **P Value for interaction** |
| --- | --- | --- | --- | --- | --- |
|  | β（95% CI） | P value | β（95% CI） | P value |  |
| Total sleep duration，min | | | | | |
| PM_2.5_ | 3.031(2.750 to 3.312) | 0.000 | 3.142(2.877 to 3.407) | 0.000 | 0.563 |
| PM_10_ | 2.790 (2.509 to 3.072) | 0.000 | 2.171 (1.919 to 2.424) | 0.000 | 0.001 |
| NO_2_ | 3.728 (3.454 to 4.002) | 0.000 | 2.839 (2.588 to 3.090) | 0.000 | 0.000 |
| O_3_ | 3.165 (2.836 to 3.493) | 0.000 | 1.367 (1.067 to 1.667) | 0.000 | 0.000 |
| SO_2_ | 2.335 (2.108 to 2.562) | 0.000 | 0.608 (0.411 to 0.805) | 0.000 | 0.000 |
| CO | 2.663 (2.355 to 2.971) | 0.000 | 2.628 (2.350 to 2.905) | 0.000 | 0.863 |
| Deep sleep duration，min | | | | | |
| PM_2.5_ | -2.268 (-2.427 to -2.110) | 0.000 | -0.739 (-0.888 to -0.589) | 0.000 | 0.000 |
| PM_10_ | -1.604 (-1.763 to -1.445) | 0.000 | -0.628 (-0.770 to -0.485) | 0.000 | 0.000 |
| NO_2_ | -2.840 (-2.994 to -2.685) | 0.000 | -2.132 (-2.274 to -1.990) | 0.000 | 0.000 |
| O_3_ | 0.707(0.521 to 0.893) | 0.000 | -1.312 (-1.482 to -1.143) | 0.000 | 0.000 |
| SO_2_ | -3.266(-3.394 to -3.139) | 0.000 | -3.927(-4.037 to -3.816) | 0.000 | 0.000 |
| CO | -5.265(-5.438 to -5.091) | 0.000 | -4.787(-4.943 to -4.631） | 0.000 | 0.000 |
| Deep sleep duration/Total sleep duration | | | | | |
| PM_2.5_ | -0.007 (-0.008 to -0.007) | 0.000 | -0.004 (-0.004 to -0.003) | 0.000 | 0.000 |
| PM_10_ | -0.006(-0.006 to -0.005) | 0.000 | -0.003 (-0.003 to -0.003) | 0.000 | 0.000 |
| NO_2_ | -0.009(-0.010 to -0.009) | 0.000 | -0.007(-0.007 to -0.007) | 0.000 | 0.000 |
| O_3_ | -2.8E-04(-6.9E-04 to 1.25E-04) | 0.174 | -0.004(-0.005 to -0.004) | 0.000 | 0.000 |
| SO_2_ | -0.010(-0.010 to -0.009) | 0.000 | -0.010(-0.010 to -0.010) | 0.000 | 0.004 |
| CO | -0.015(-0.015 to -0.014) | 0.000 | -0.014(-0.014 to -0.013) | 0.000 | 0.000 |
| Light sleep duration，min | | | | | |
| PM_2.5_ | 5.299 (5.040 to 5.558) | 0.000 | 3.881 (3.636 to 4.126) | 0.000 | 0.000 |
| PM_10_ | 4.394 (4.135 to 4.654) | 0.000 | 2.799(2.566 to 3.032) | 0.000 | 0.000 |
| NO_2_ | 6.568 (6.315 to 6.821) | 0.000 | 4.971 (4.739 to 5.202) | 0.000 | 0.000 |
| O_3_ | 2.458 (2.154 to 2.761) | 0.000 | 2.679 (2.402 to 2.956) | 0.000 | 0.284 |
| SO_2_ | 5.601 (5.392 to 5.810) | 0.000 | 4.535 (4.353 to 4.716) | 0.000 | 0.000 |
| CO | 7.928 (7.644 to 8.211) | 0.000 | 7.415 (7.159 to 7.670) | 0.000 | 0.006 |
| Deep sleep duration/ Light sleep duration | | | | | |
| PM_2.5_ | -0.016 (-0.016 to -0.015) | 0.000 | -0.010 (-0.010 to -0.009) | 0.000 | 0.000 |
| PM_10_ | -0.012(-0.013 to -0.011) | 0.000 | -0.008(-0.008 to -0.007) | 0.000 | 0.000 |
| NO_2_ | -0.020(-0.020 to -0.019) | 0.000 | -0.016 (-0.017 to -0.015) | 0.000 | 0.000 |
| O_3_ | -0.001 (-0.002 to -4.8E-04) | 0.003 | -0.009(-0.010 to -0.009) | 0.000 | 0.000 |
| SO_2_ | -0.019(-0.020 to -0.019) | 0.000 | -0.022(-0.022 to -0.021) | 0.000 | 0.000 |
| CO | -0.029(-0.030 to -0.029) | 0.000 | -0.030(-0.031 to -0.029) | 0.000 | 0.434 |
| Times of WASO per hour of sleep | | | | | |
| PM_2.5_ | -0.004(-0.005 to -0.003) | 0.000 | -0.007(-0.007 to -0.006) | 0.000 | 0.000 |
| PM_10_ | -0.004(-0.004 to -0.003) | 0.000 | -0.005(-0.005 to -0.004) | 0.000 | 0.013 |
| NO_2_ | -3.4E-04(-0.001 to 3.7E-04) | 0.348 | -0.008(-0.008 to -0.007) | 0.000 | 0.000 |
| O_3_ | -0.001(-0.002 to -2.7E-04) | 0.010 | 0.001(2.83E-05 to 0.002) | 0.042 | 0.001 |
| SO_2_ | -0.003(-0.003 to -0.002) | 0.000 | -3.9E-04(-0.001 to 2.42E-04) | 0.226 | 0.000 |
| CO | -0.004(-0.005 to -0.003) | 0.000 | -0.006 (-0.007 to -0.005) | 0.000 | 0.002 |
| Duration of WASO/ Total sleep duration | | | | | |
| PM_2.5_ | -6.7E-04(-8.4E-04 to -5.1E-04) | 0.000 | -0.001 (-0.002 to -0.001) | 0.000 | 0.000 |
| PM_10_ | -4.8E-04(-6.5E-04 to -3.2E-04) | 0.000 | -0.001 (-0.001 to -0.001) | 0.000 | 0.000 |
| NO_2_ | -4.4E-04(-0.001 to -2.8E-04) | 0.000 | -8.4E-04(-9.8E-04 to -6.9E-04) | 0.000 | 0.000 |
| O_3_ | -4.4E-04 (-6.4E-04 to -2.5E-04) | 0.000 | 2.03E-04(2.8E-05 to 3.79E-04) | 0.023 | 0.000 |
| SO_2_ | -0.001(-0.001 to -0.001) | 0.000 | -0.001(-0.001 to -0.001) | 0.000 | 0.054 |
| CO | -0.001(-0.001 to -0.001) | 0.000 | -0.002(-0.002 to -0.002) | 0.000 | 0.000 |

Note: data are β (95%CI). β indicates partial regression coefficient. Estimates were associated with per 1-interquartile range increase in concentration of each pollutant (PM_2.5_, 17.0µg/m^3^; PM_10_, 31.2µg/m^3^; NO_2_, 10.3µg/m^3^; O_3_ 13.1µg/m^3^; SO_2_ 7.1µg/m^3^; CO 0.3 mg/m^3^). Adjusted for age, sex, BMI, city development level, altitude, season, and the type of night.

Abbreviations: CI, confidence intervals; CO, carbon monoxide; NO_2_, nitrogen dioxide; O_3_, ozone; PM_2.5_, particulate matter with aerodynamic diameter ≤2.5 μm; PM_10_, particulate matter with aerodynamic diameter ≤10 μm; SO_2_, sulfur dioxide; WASO: wake after sleep onset.

**Supplementary Table S4: Adjusted subgroup analysis of the associations between sleep parameters and long-term exposures to ambient air pollutants by sleep duration**

|  | **< 7 hours** | | **≥ 7 hours** | | **P Value for interaction** |
| --- | --- | --- | --- | --- | --- |
|  | β（95% CI） | P value | β（95% CI） | P value |  |
| Total sleep duration, min | | | | | |
| PM_2.5_ | 0.689 (0.514 to 0.863) | 0.000 | 0.940 (0.771 to 1.110) | 0.000 | 0.037 |
| PM_10_ | 0.515 (0.345 to 0.684) | 0.000 | 0.633 (0.468 to 0.799) | 0.000 | 0.310 |
| NO_2_ | 0.678 (0.512 to 0.844) | 0.000 | 1.084 (0.920 to 1.248) | 0.000 | 0.000 |
| O_3_ | 0.577 (0.376 to 0.779) | 0.000 | 0.850(0.656 to 1.044) | 0.000 | 0.052 |
| SO_2_ | 0.462 (0.328 to 0.596) | 0.000 | 0.539 (0.408 to 0.669) | 0.000 | 0.389 |
| CO | 0.954 (0.765 to 1.143) | 0.000 | 0.825 (0.646 to 1.003) | 0.000 | 0.309 |
| Deep sleep duration, min | | | | | |
| PM_2.5_ | -1.607 (-1.755 to -1.460) | 0.000 | -2.339 (-2.483 to -2.195) | 0.000 | 0.000 |
| PM_10_ | -1.036(-1.180 to -0.892) | 0.000 | -1.943 (-2.084 to -1.802) | 0.000 | 0.000 |
| NO_2_ | -2.758 (-2.899 to -2.617) | 0.000 | -3.249(-3.388 to -3.110) | 0.000 | 0.000 |
| O_3_ | -1.161 (-1.332 to -0.990) | 0.000 | -0.308 (-0.473 to -0.143) | 0.000 | 0.000 |
| SO_2_ | -3.524(-3.638 to -3.410) | 0.000 | -4.135(-4.246 to -4.024) | 0.000 | 0.000 |
| CO | -5.033 (-5.194 to -4.873) | 0.000 | -5.748 (-5.899 to -5.596) | 0.000 | 0.000 |
| Deep sleep duration/Total sleep duration | | | | | |
| PM_2.5_ | -0.005 (-0.006 to -0.005) | 0.000 | -0.006(-0.006 to -0.005) | 0.000 | 0.243 |
| PM_10_ | -0.004(-0.004 to -0.003) | 0.000 | -0.004(-0.005 to -0.004) | 0.000 | 0.001 |
| NO_2_ | -0.008(-0.008 to -0.008) | 0.000 | -0.008(-0.008 to -0.007) | 0.000 | 0.213 |
| O_3_ | -0.003(-0.004 to -0.003) | 0.000 | -0.001(-0.002 to -0.001) | 0.000 | 0.000 |
| SO_2_ | -0.010(-0.011 to -0.010) | 0.000 | -0.009(-0.009 to -0.009) | 0.000 | 0.000 |
| CO | -0.015(-0.016 to -0.015) | 0.000 | -0.013(-0.013 to -0.012) | 0.000 | 0.000 |
| Light sleep duration，min | | | | | |
| PM_2.5_ | 2.296 (2.102 to 2.490) | 0.000 | 3.279 (3.090 to 3.468) | 0.000 | 0.000 |
| PM_10_ | 1.551 (1.362 to 1.739) | 0.000 | 2.576 (2.392 to 2.761) | 0.000 | 0.000 |
| NO_2_ | 3.437 (3.252 to 3.622) | 0.000 | 4.333 (4.150 to 4.516) | 0.000 | 0.000 |
| O_3_ | 1.738 (1.512 to 1.963) | 0.000 | 1.158 (0.942 to 1.375) | 0.000 | 0.000 |
| SO_2_ | 3.986 (3.837 to 4.135) | 0.000 | 4.674 (4.529 to 4.819) | 0.000 | 0.000 |
| CO | 5.987 (5.777 to 6.198) | 0.000 | 6.572 (6.374 to 6.771) | 0.000 | 0.000 |
| Deep sleep duration/ Light sleep duration | | | | | |
| PM_2.5_ | -0.012 (-0.013 to -0.011) | 0.000 | -0.012 (-0.013 to -0.011) | 0.000 | 0.665 |
| PM_10_ | -0.008 (-0.009 to -0.008) | 0.000 | -0.010(-0.010 to -0.009) | 0.000 | 0.006 |
| NO_2_ | -0.017 (-0.018 to -0.017) | 0.000 | -0.017(-0.017 to -0.016) | 0.000 | 0.118 |
| O_3_ | -0.008(-0.009 to -0.007) | 0.000 | -0.003(-0.004 to -0.002) | 0.000 | 0.000 |
| SO_2_ | -0.023(-0.023 to -0.022) | 0.000 | -0.018 (-0.019 to -0.018) | 0.000 | 0.000 |
| CO | -0.033 (-0.034 to -0.032) | 0.000 | -0.026(-0.027 to -0.025) | 0.000 | 0.000 |
| Times of WASO per hour of sleep | | | | | |
| PM_2.5_ | -0.004 (-0.005 to -0.003) | 0.000 | -0.007 (-0.008 to -0.006) | 0.000 | 0.000 |
| PM_10_ | -0.003(-0.003 to -0.002) | 0.000 | -0.006 (-0.007 to -0.005) | 0.000 | 0.000 |
| NO_2_ | -0.005 (-0.006 to -0.005) | 0.000 | -0.004 (-0.004 to -0.003) | 0.000 | 0.001 |
| O_3_ | 0.003 (0.002 to 0.004) | 0.000 | -0.003 (-0.004 to -0.002) | 0.000 | 0.000 |
| SO_2_ | 6.16E-04( -6.7E-05 to 0.001) | 0.077 | -0.003 (-0.004 to -0.003) | 0.000 | 0.000 |
| CO | -0.005 (-0.005 to -0.004) | 0.000 | -0.006(-0.007 to -0.005) | 0.000 | 0.005 |
| Duration of WASO/ Total sleep duration | | | | | |
| PM_2.5_ | -5.9E-04(-7.6E-04 to -4.3E-04) | 0.000 | -0.001(-0.001 to -0.001) | 0.000 | 0.000 |
| PM_10_ | -2.6E-04(-4.1E-04 to -1.0E-04) | 0.001 | -0.001(-0.001 to -0.001) | 0.000 | 0.000 |
| NO_2_ | -2.7E-04(-4.2E-04 to -1.1E-04) | 0.001 | -8.6E-04(-0.001 to -7.1E-04) | 0.000 | 0.000 |
| O_3_ | 7.79E-04 (5.92E-04 to 9.65E-04) | 0.000 | -7.6E-04 (-9.4E-04 to -5.8E-04) | 0.000 | 0.000 |
| SO_2_ | -9.8E-04(-0.001 to -8.5E-04) | 0.000 | -0.001 (-0.001 to -0.001) | 0.000 | 0.000 |
| CO | -0.001 (-0.002 to -0.001) | 0.000 | -0.002 (-0.002 to -0.002) | 0.000 | 0.011 |

Note: data are β (95%CI). β indicates partial regression coefficient. Estimates were associated with per 1-interquartile range increase in concentration of each pollutant (PM_2.5_, 17.0µg/m^3^; PM_10_, 31.2µg/m^3^; NO_2_, 10.3µg/m^3^; O_3_ 13.1µg/m^3^; SO_2_ 7.1µg/m^3^; CO 0.3 mg/m^3^). Adjusted for age, sex, BMI, city development level, altitude, season, and the type of night.

Abbreviations: CI, confidence intervals; CO, carbon monoxide; NO_2_, nitrogen dioxide; O_3_, ozone; PM_2.5_, particulate matter with aerodynamic diameter ≤2.5 μm; PM_10_, particulate matter with aerodynamic diameter ≤10 μm; SO_2_, sulfur dioxide; WASO: wake after sleep onset.

**Supplementary Table S5: Adjusted subgroup analysis of the associations between sleep parameters and long-term exposures to ambient air pollutants by season ^a^**

|  | **Warm seasons** | | **Cold seasons** | | **P Value for interaction** |
| --- | --- | --- | --- | --- | --- |
|  | β（95% CI） | P value | β（95% CI） | P value |  |
| Total sleep duration，min | | | | | |
| PM_2.5_ | 1.907 (1.631 to 2.184) | 0.000 | 4.203 (3.935 to 4.471) | 0.000 | 0.000 |
| PM_10_ | 1.209 (0.940 to 1.477) | 0.000 | 3.619 (3.357 to 3.882) | 0.000 | 0.000 |
| NO_2_ | 3.383 (3.117 to 3.649) | 0.000 | 3.108 (2.850 to 3.365) | 0.000 | 0.118 |
| O_3_ | 2.585 (2.271 to 2.898) | 0.000 | 1.773 (1.459 to 2.086) | 0.000 | 0.000 |
| SO_2_ | 0.444 (0.228 to 0.659) | 0.000 | 2.120 (1.915 to 2.325) | 0.000 | 0.000 |
| CO | 1.140 (0.843 to 1.438) | 0.000 | 4.022 (3.737 to 4.308) | 0.000 | 0.000 |
| Deep sleep duration，min | | | | | |
| PM_2.5_ | -1.240 (-1.396 to -1.084) | 0.000 | -1.670 (-1.822 to -1.518) | 0.000 | 0.000 |
| PM_10_ | -1.249 (-1.401 to -1.097) | 0.000 | -0.901 (-1.049 to -0.753) | 0.000 | 0.001 |
| NO_2_ | -2.260(-2.410 to -2.110) | 0.000 | -2.655 (-2.801 to -2.510) | 0.000 | 0.000 |
| O_3_ | -1.317 (-1.494 to -1.140) | 0.000 | 0.550 (0.373 to 0.727) | 0.000 | 0.000 |
| SO_2_ | -3.822 (-3.943 to -3.700) | 0.000 | -3.495 (-3.610 to -3.379) | 0.000 | 0.000 |
| CO | -4.863 (-5.030 to -4.696) | 0.000 | -5.141(-5.301 to -4.980) | 0.000 | 0.014 |
| Deep sleep duration/Total sleep duration | | | | | |
| PM_2.5_ | -0.004 (-0.005 to -0.004) | 0.000 | -0.007 (-0.007 to -0.006) | 0.000 | 0.000 |
| PM_10_ | -0.004 (-0.004 to -0.004) | 0.000 | -0.004 (-0.005 to -0.004) | 0.000 | 0.221 |
| NO_2_ | -0.008(-0.008 to -0.008) | 0.000 | -0.008 (-0.009 to -0.008) | 0.000 | 0.080 |
| O_3_ | -0.005 (-0.005 to -0.005) | 0.000 | 1.66E-05(-3.7E-04 to 4.04E-04) | 0.933 | 0.000 |
| SO_2_ | -0.010 (-0.010 to -0.010) | 0.000 | -0.010 (-0.010 to -0.009) | 0.000 | 0.174 |
| CO | -0.013 (-0.013 to -0.013) | 0.000 | -0.015 (-0.015 to -0.015) | 0.000 | 0.000 |
| Light sleep duration，min | | | | | |
| PM_2.5_ | 3.147 (2.892 to3.402) | 0.000 | 5.873 (5.625 to 6.121) | 0.000 | 0.000 |
| PM_10_ | 2.458 (2.210 to 2.706) | 0.000 | 4.521 (4.279 to 4.762) | 0.000 | 0.000 |
| NO_2_ | 5.643 (5.398 to 5.888) | 0.000 | 5.763 (5.526 to 6.001) | 0.000 | 0.462 |
| O_3_ | 3.902 (3.613 to 4.191) | 0.000 | 1.223 (0.934 to 1.512) | 0.000 | 0.000 |
| SO_2_ | 4.266 (4.067 to 4.464) | 0.000 | 5.615(5.426 to 5.804) | 0.000 | 0.000 |
| CO | 6.003 (5.730 to 6.277) | 0.000 | 9.163 (8.900 to 9.426) | 0.000 | 0.000 |
| Deep sleep duration/ Light sleep duration | | | | | |
| PM_2.5_ | -0.010 (-0.011 to -0.010) | 0.000 | -0.014(-0.015 to -0.014) | 0.000 | 0.000 |
| PM_10_ | -0.009 (-0.010 to -0.008) | 0.000 | -0.010 (-0.011 to -0.009) | 0.000 | 0.185 |
| NO_2_ | -0.017 (-0.018 to -0.016) | 0.000 | -0.018 (-0.019 to -0.017) | 0.000 | 0.047 |
| O_3_ | -0.011 (-0.012 to -0.010) | 0.000 | -5.7E-04(-0.001 to 2.75E-04) | 0.186 | 0.000 |
| SO_2_ | -0.021 (-0.021 to -0.020) | 0.000 | -0.020(-0.021 to -0.020) | 0.000 | 0.121 |
| CO | -0.028 (-0.028 to -0.027) | 0.000 | -0.032 (-0.032 to -0.031) | 0.000 | 0.000 |
| Times of WASO per hour of sleep | | | | | |
| PM_2.5_ | -0.005 (-0.006 to -0.004) | 0.000 | -0.006 (-0.006 to -0.005) | 0.000 | 0.179 |
| PM_10_ | -0.004(-0.005 to -0.003) | 0.000 | -0.005(-0.005 to -0.004) | 0.000 | 0.105 |
| NO_2_ | -0.006(-0.006 to -0.005) | 0.000 | -0.004 (-0.004 to -0.003) | 0.000 | 0.000 |
| O_3_ | 5.22E-04(-3.1E-04 to 1.35E-04) | 0.216 | -6.1E-04(-0.001 to 1.6E-04) | 0.120 | 0.046 |
| SO_2_ | -0.001 (-0.002 to -4.5E-04) | 0.002 | -0.002 (-0.002 to -9.2E-04) | 0.000 | 0.472 |
| CO | -0.006(-0.007 to -0.005) | 0.000 | -0.005 (-0.006 to -0.004) | 0.000 | 0.084 |
| Duration of WASO/ Total sleep duration | | | | | |
| PM_2.5_ | -0.001 (-0.001 to -9.7E-04) | 0.000 | -9.8E-04(-0.001 to -8.2E-04) | 0.000 | 0.189 |
| PM_10_ | -0.001 (-0.00116 to -0.00085) | 0.000 | -7.2E-04(-8.7E-04 to -5.6E-04) | 0.000 | 0.008 |
| NO_2_ | -8.5E-04(-0.001 to -7.0E-04) | 0.000 | -5.0E-04(-6.0E-04 to -3.4E-04) | 0.000 | 0.001 |
| O_3_ | -1.5E-04(-3.3E-04 to 3.35E-05) | 0.109 | -1.7E-05(-2.0E-04 to 1.66E-04) | 0.857 | 0.308 |
| SO_2_ | -0.001(-0.001 to -0.001) | 0.000 | -0.001 (-0.001 to -9.2E-04) | 0.000 | 0.000 |
| CO | -0.002 (-0.002 to -0.002) | 0.000 | -0.002 (-0.002 to -0.001) | 0.000 | 0.116 |

^a^ Warm season include spring and summer, from March to August. Cold seasons include autumn and winter, from September to February.

Note: data are β (95%CI). β indicates partial regression coefficient. Estimates were associated with per 1-interquartile range increase in concentration of each pollutant (PM_2.5_, 17.0µg/m^3^; PM_10_, 31.2µg/m^3^; NO_2_, 10.3µg/m^3^; O_3_ 13.1µg/m^3^; SO_2_ 7.1µg/m^3^; CO 0.3 mg/m^3^). Adjusted for age, sex, BMI, city development level, altitude, season, and the type of night.

Abbreviations: CI, confidence intervals; CO, carbon monoxide; NO_2_, nitrogen dioxide; O_3_, ozone; PM_2.5_, particulate matter with aerodynamic diameter ≤2.5 μm; PM_10_, particulate matter with aerodynamic diameter ≤10 μm; SO_2_, sulfur dioxide; WASO: wake after sleep onset.

**Supplementary Figure S1 (a-g): Stratified analysis 1—Effect of short-term pollutant exposure on sleep parameters.**

Use the method of extracting records every 7 days intervals. Data are β (95%CI). β indicates partial regression coefficient. Estimates were associated with per 1-interquartile range increase in concentration of each pollutant. Adjusted for age, sex, BMI, city development level, altitude, season, and the type of night. * p < 0.05.

Abbreviations: CI, confidence intervals; CO, carbon monoxide; NO_2_, nitrogen dioxide; O_3_, ozone; PM_2.5_, particulate matter with aerodynamic diameter ≤2.5μm; PM_10_, particulate matter with aerodynamic diameter ≤10μm; SO_2_, sulfur dioxide; WASO: wake after sleep onset.


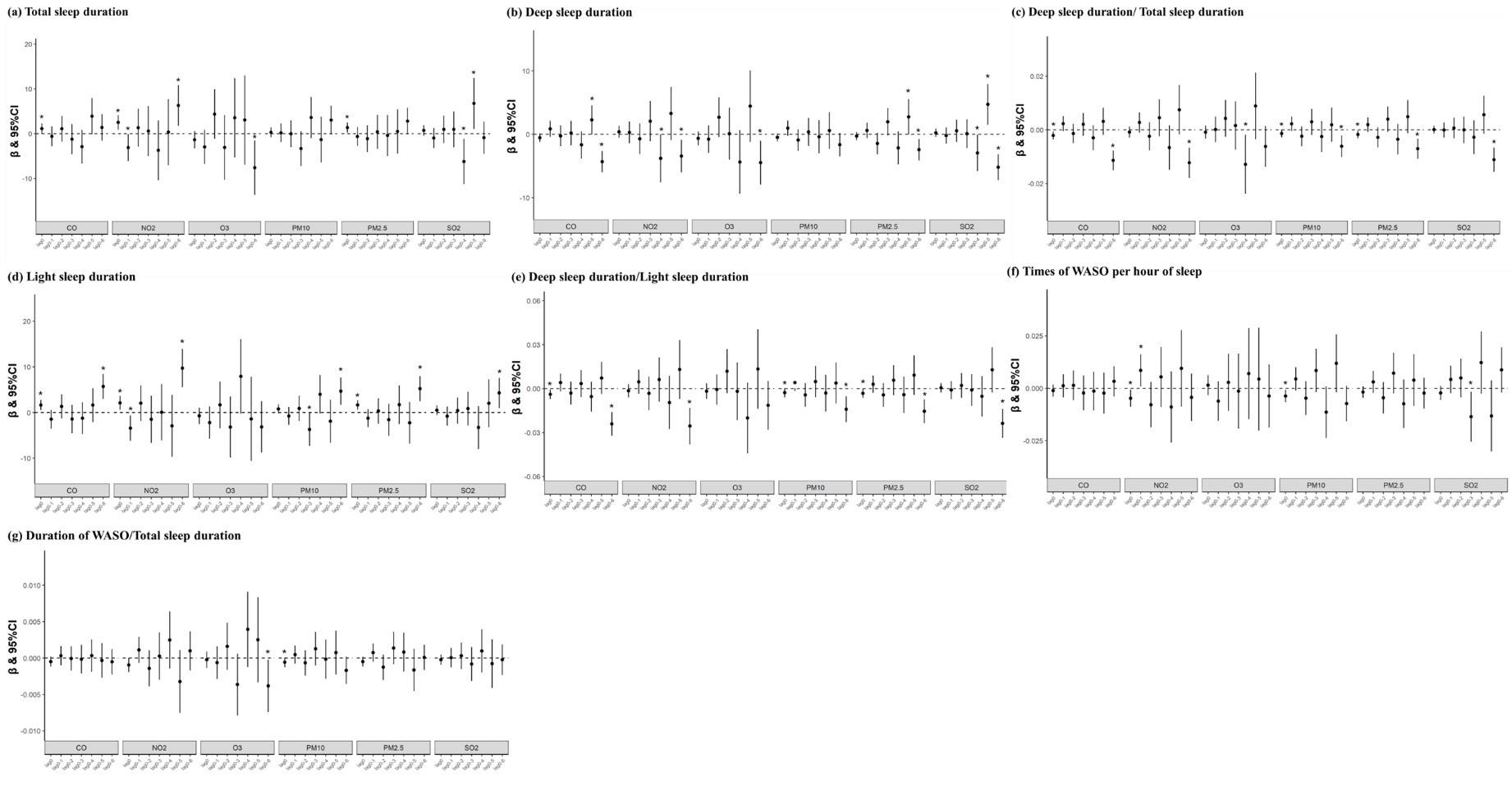


**Supplementary Figure S2 (a-g): Stratified analysis 2—Effect of short-term pollutant exposure on sleep parameters.**

Use the method of calculating the average value of the first 7 records of each data set. Data are β (95%CI). β indicates partial regression coefficient. Estimates were associated with per 1-interquartile range increase in concentration of each pollutant. Adjusted for age, sex, BMI, city development level, altitude, season, and the type of night. * p < 0.05.

Abbreviations: CI, confidence intervals; CO, carbon monoxide; NO_2_, nitrogen dioxide; O_3_, ozone; PM_2.5_, particulate matter with aerodynamic diameter ≤2.5μm; PM_10_, particulate matter with aerodynamic diameter ≤10 μm; SO_2_, sulfur dioxide; WASO: wake after sleep onset.


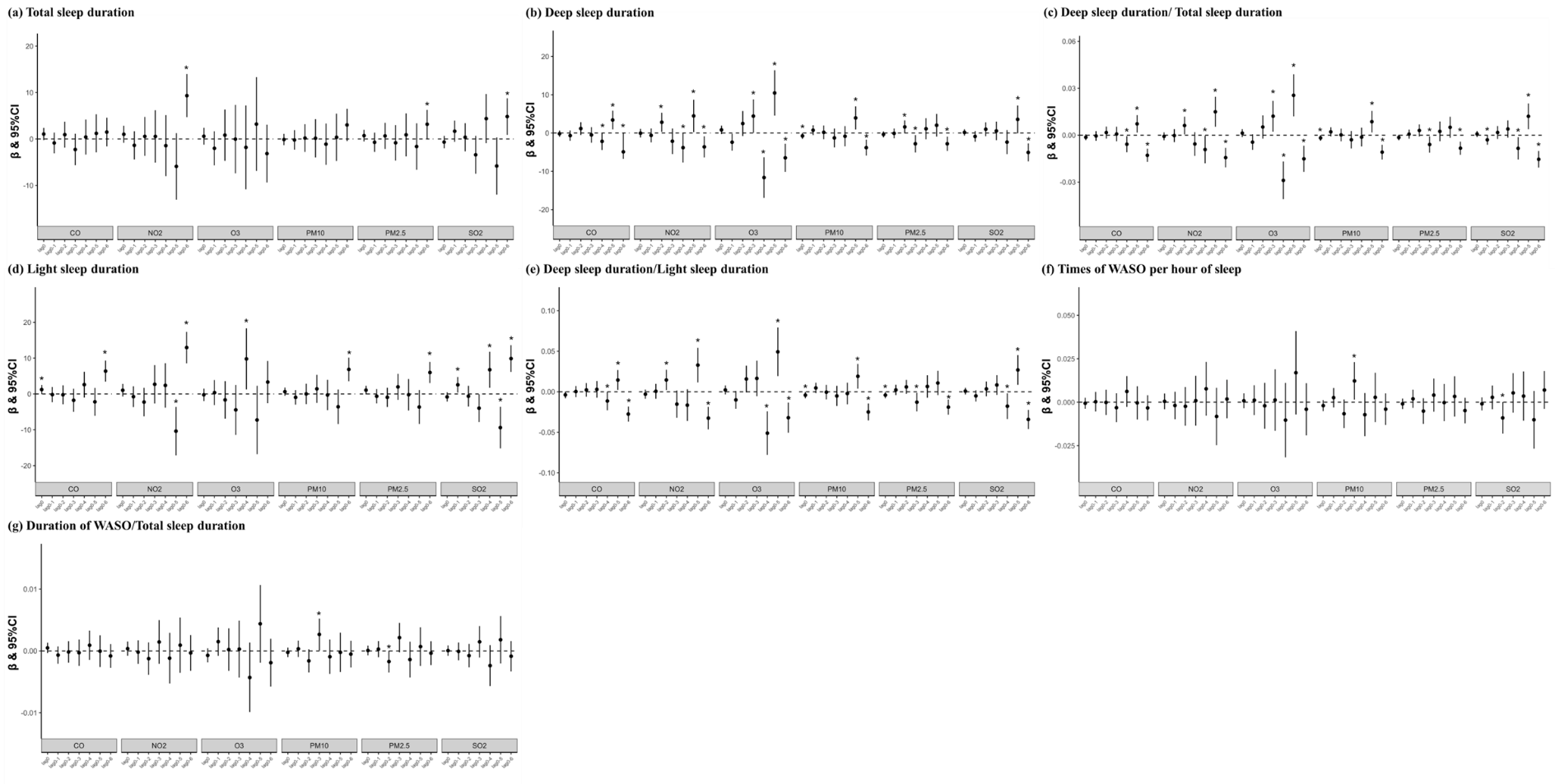


**Supplementary Table S6: Associations between sleep parameters and long-term exposures to ambient air pollutants when adjusting for year, month, and day of week**

|  | PM_2.5_ | | PM_10_ | | NO_2_ | | O_3_ | | SO_2_ | | CO | |
| --- | --- | --- | --- | --- | --- | --- | --- | --- | --- | --- | --- | --- |
|  | β  （95% CI） | P value | β  （95% CI） | P value | β  （95% CI） | P value | β  （95% CI） | P value | β  （95% CI） | P value | β  （95% CI） | p value |
| Total sleep duration，min | 3.082 (2.885 to 3.279) | 0.000 | 2.409 (2.215 to 2.602) | 0.000 | 3.212 (3.016 to 3.408) | 0.000 | 2.106 (1.881 to 2.330) | 0.000 | 1.339 (1.182 to 1.497) | 0.000 | 2.662 (2.447 to 2.876) | 0.000 |
| Deep sleep duration, min | -1.466 (-1.578 to -1.355) | 0.000 | -1.057 (-1.166 to -0.947) | 0.000 | -2.453(-2.564 to -2.342) | 0.000 | -0.370 (-0.497 to -0.243) | 0.000 | -3.645 (-3.734 to -3.556) | 0.000 | -5.021 (-5.142 to -4.900) | 0.000 |
| Deep sleep duration/Total sleep duration | -0.006(-0.006 to -0.005) | 0.000 | -0.004 (-0.004 to -0.004) | 0.000 | -0.008 (-0.008 to -0.008) | 0.000 | -0.002 (-0.003 to -0.002) | 0.000 | -0.010 (-0.010 to -0.010) | 0.000 | -0.014 (-0.014 to -0.014) | 0.000 |
| Light sleep duration，min | 4.548 (4.366 to  4.730) | 0.000 | 3.465 (3.287 to  3.644) | 0.000 | 5.665 (5.484 to 5.845) | 0.000 | 2.476 (2.269 to 2.683) | 0.000 | 4.984 (4.839 to 5.130） | 0.000 | 7.682 (7.485 to 7.880) | 0.000 |
| Deep sleep duration/ Light sleep duration | -0.012 (-0.013 to -0.012) | 0.000 | -0.009 (-0.010 to -0.009) | 0.000 | -0.017(-0.018 to -0.017) | 0.000 | -0.006(-0.006 to -0.005) | 0.000 | -0.021 (-0.021 to-0.020) | 0.000 | -0.030 (-0.03 to -0.029) | 0.000 |
| Times of WASO per hour of sleep | -0.005 (-0.006 to -0.005) | 0.000 | -0.004 (-0.005 to -0.004) | 0.000 | -0.004 (-0.005 to-0.004) | 0.000 | 8.04E-05(-5.0E-04 to 6.6E-04) | 0.784 | -0.001 (-0.002 to-0.001) | 0.000 | -0.005 (-0.006 to-0.004) | 0.000 |
| Duration of WASO/ Total sleep duration | -0.001 (-0.001 to -0.001) | 0.000 | -8.7E-04(-9.8E-04 to -7.5E-04) | 0.000 | -6.7E-04(-7.9E-04 to -5.6E-04) | 0.000 | -8.53E-05(-2.2E-04 to 4.62E-05) | 0.203 | -0.001 (-0.001 to -0.001) | 0.000 | -0.002 (-0.002 to -0.002) | 0.000 |

Note: data are β (95%CI). β indicates partial regression coefficient. Estimates were associated with per 1-interquartile range increase in concentration of each pollutant (PM_2.5_, 17.0µg/m^3^; PM_10_, 31.2µg/m^3^; NO_2_, 10.3µg/m^3^; O_3_ 13.1µg/m^3^; SO_2_ 7.1µg/m^3^; CO 0.3 mg/m^3^). Adjusted for age, sex, BMI, city development level, altitude, year, month, and day of week.

Abbreviations: CI, confidence intervals; CO, carbon monoxide; NO_2_, nitrogen dioxide; O_3_, ozone; PM_2.5_, particulate matter with aerodynamic diameter ≤2.5 μm; PM_10_, particulate matter with aerodynamic diameter ≤10 μm; SO_2_, sulfur dioxide; WASO: wake after sleep onset.

**Supplementary Table S7: Associations between sleep parameters and short-term exposure to ambient air pollutants when adjusting for year, month, and day of week**

|  | PM_2.5_ | | PM_10_ | | NO_2_ | | O_3_ | | SO_2_ | | CO | |
| --- | --- | --- | --- | --- | --- | --- | --- | --- | --- | --- | --- | --- |
|  | β（95% CI） | P value | β（95% CI） | P value | β（95% CI） | P value | β（95% CI） | P value | β（95% CI） | P value | β（95% CI） | P value |
| Total sleep duration，min | | | | | | | | | | | | |
| lag0 | 0.357 (-0.035 to 0.748) | 0.075 | -0.180(-0.567 to 0.207) | 0.362 | 0.642 (0.051 to 1.231) | 0.033 | -2.327 (-3.034 to -1.619) | 0.000 | -0.419 (-0.823 to -0.014) | 0.042 | 0.733 (0.320 to 1.146) | 0.001 |
| lag0-1 | -0.016 (-0.794 to  0.762) | 0.967 | 0.177 (-0.591 to  0.945) | 0.651 | -0.448 (-1.563 to 0.668) | 0.432 | 0.763 (-0.664 to 2.190) | 0.295 | 0.431(-0.388 to 1.249) | 0.303 | -0.311 (-1.122 to 0.499) | 0.452 |
| lag0-2 | -0.628 (-1.731 to 0.475) | 0.264 | -0.485 (-1.585 to 0.616) | 0.388 | 0.865 (-0.714 to 2.444) | 0.283 | -0.620 (-2.707 to 1.467) | 0.560 | 0.148 (-1.006 to 1.303) | 0.801 | -0.600 (-1.655 to 0.455) | 0.265 |
| lag0-3 | -0.964 (-2.378 to 0.450) | 0.182 | -0.996 (-2.458 to 0.467) | 0.182 | -3.375 (-5.463 to -1.286) | 0.002 | 1.625 (-1.111 to 4.360) | 0.244 | -1.538 (-3.034 to -0.043) | 0.044 | -0.461 (-1.714 to 0.792) | 0.471 |
| lag0-4 | 1.683 (-0.037 to 3.403) | 0.055 | 1.404 (-0.340 to 3.148) | 0.115 | 1.805 (-0.722 to 4.332) | 0.161 | -1.680 (-5.026 to 1.667) | 0.325 | 0.319 (-1.583 to 2.222) | 0.742 | -0.653 (-2.054 to 0.749) | 0.361 |
| lag0-5 | -1.544 (-3.414 to 0.327) | 0.106 | -1.329 (-3.265 to 0.607) | 0.178 | -2.498(-5.306 to 0.310) | 0.081 | 4.304 (0.529 to 8.079) | 0.025 | -0.378 (-2.5483 to 1.792) | 0.733 | 1.267 (-0.249 to 2.783) | 0.101 |
| lag0-6 | 2.887 (1.777 to 3.998) | 0.000 | 3.027 (1.826 to 4.229) | 0.000 | 5.517 (3.795 to 7.239) | 0.000 | -3.286 (-5.579 to -0.992) | 0.000 | 2.319 (0.953 to 3.685) | 0.001 | 1.794(0.695 to 2.892) | 0.001 |
| Deep sleep duration, min | | | | | | | | | | | | |
| lag0 | -0.305 (-0.527 to -0.084) | 0.007 | -0.384 (-0.602 to -0.165) | 0.001 | -0.232 (-0.564 to 0.101) | 0.173 | -0.677 (-1.077 to -0.278) | 0.001 | -0.073 (-0.301 to 0.155) | 0.531 | -0.305(-0.538 to -0.072) | 0.010 |
| lag0-1 | -0.099 (-0.538 to 0.341) | 0.660 | 0.051 (-0.382 to 0.485) | 0.817 | -0.175 (-0.805 to 0.455) | 0.585 | -0.290 (-1.096 to 0.516) | 0.481 | -0.296 (-0.758 to 0.165) | 0.208 | -0.183 (-0.640 to 0.274) | 0.433 |
| lag0-2 | 0.237 (-0.386 to 0.860) | 0.457 | 0.118 (-0.503 to 0.739) | 0.710 | 0.3433 (-0.548 to 1.235) | 0.450 | 0.738 (-0.441 to 1.917) | 0.220 | 0.135 (-0.515 to 0.786) | 0.683 | 0.242 (-0.353 to 0.838) | 0.425 |
| lag0-3 | -0.048(-0.846 to 0.751) | 0.907 | -0.247 (-1.073 to 0.579) | 0.558 | 0.329(-0.850 to 1.508) | 0.584 | 0.707(-0.838 to 2.252) | 0.370 | 0.229 (-0.614 to  1.072) | 0.594 | -0.329 (-1.036 to 0.378) | 0.361 |
| lag0-4 | -0.456 (-1.428 to 0.515) | 0.357 | -0.090 (-1.075 to 0.895) | 0.858 | -1.932 (-3.359 to -0.505) | 0.008 | -1.735 (-3.625 to 0.155) | 0.072 | -0.813 (-1.885 to 0.260) | 0.138 | -0.278(-1.068 to 0.513) | 0.491 |
| lag0-5 | 1.238 (0.181 to 2.295) | 0.022 | 0.861 (-0.233 to 1.954) | 0.123 | 3.047 (1.461 to 4.632) | 0.000 | 2.832 (0.700 to 4.965) | 0.009 | 1.771 (0.547 to 2.994 | 0.005 | 1.219(0.3642 to 2.074) | 0.005 |
| lag0-6 | -1.771 (-2.398 to -1.143) | 0.000 | -2.067 (-2.746 to -1.388) | 0.000 | -4.451 (-5.423 to -3.479) | 0.000 | -3.642 (-4.937 to -2.346) | 0.000 | -3.915 (-4.685 to -3.145) | 0.000 | -4.279 (-4.899 to -3.660) | 0.000 |
| Deep sleep duration/Total sleep duration | | | | | | | | | | | | |
| lag0 | -0.001 (-0.001 to -0.001) | 0.000 | -0.001 (-0.001 to -3.47E-04) | 0.001 | -0.001(-0.002 to -1.8E-04) | 0.014 | -3.5E-04(-0.001 to 0.001) | 0.434 | 1.53E-05(-4.84E-04 to 0.001) | 0.952 | -0.001 (-0.002 to -6.85E-04) | 0.000 |
| lag0-1 | -1.66E-04(-0.001 to 7.97E-04) | 0.735 | 1.12E-04 (-0.001 to 0.001) | 0.817 | -2.3E-04(-0.002 to 0.001) | 0.744 | -0.001 (-0.003 to 0.001) | 0.191 | 0.001(-0.002 to  1.8E-04) | 0.107 | -2.1E-04(-0.001 to 0.001) | 0.676 |
| lag0-2 | 0.001 (-3.8E-04 to 0.002) | 0.158 | 4.49E-04 (-0.001 to 0.002) | 0.518 | 4.6E-04(-0.002 to 0.002) | 0.641 | 0.003 (-1.85E-05 to 0.005) | 0.052 | 1.77E-05(-0.001 to 0.001) | 0.981 | 0.001 (-4.6E-04 to 0.002) | 0.206 |
| lag0-3 | 1.41E-04(-0.002 to 0.002) | 0.874 | -8.77E-05(-0.002 to 0.002) | 0.924 | 0.002 (1.6E-04 to 0.005) | 0.066 | 2.5E-04(-0.003 to 0.004) | 0.885 | 0.002 (-1.64E-04 to 0.004) | 0.074 | -4.6E-04 (-0.002 to 0.001) | 0.562 |
| lag0-4 | -0.002 (-0.004 to  2.97E-04) | 0.092 | -0.001 (-0.003 to 0.001) | 0.362 | -0.006(-0.009 to -0.002) | 0.001 | -0.003 (-0.007 to 0.001) | 0.138 | -0.002 (-0.005 to  1.1E-04) | 0.062 | -4.1E-04 (-0.002 to 0.001) | 0.642 |
| lag0-5 | 0.004(0.002 to 0.006) | 0.001 | 0.003 (8.33E-04 to 0.006) | 0.008 | 0.009 (0.005 to 0.012) | 0.000 | 0.005(2.89E-05 to 0.009) | 0.049 | 0.004 (0.002 to 0.007) | 0.001 | 0.002 (0.001 to 0.004) | 0.012 |
| lag0-6 | -0.006(-0.007 to -0.005) | 0.000 | -0.007 (-0.009 to -0.006) | 0.000 | -0.014 (-0.016 to -0.012) | 0.000 | -0.007(-0.010 to -0.004) | 0.000 | -0.011 (-0.013 to -0.009) | 0.000 | -0.012 (-0.013 to -0.010) | 0.000 |
| Light sleep duration，min | | | | | | | | | | | | |
| lag0 | 0.662(0.300 to 1.024) | 0.000 | 0.204 (-0.153 to 0.561) | 0.264 | 0.874 (0.330 to 1.417) | 0.002 | -1.650 (-2.303 to -0.996) | 0.000 | -0.346 (-0.719 to 0.027) | 0.069 | 1.038(0.657 to 1.419) | 0.000 |
| lag0-1 | 0.082 (-0.636 to 0.800) | 0.822 | 0.126 (-0.583 to 0.835) | 0.728 | -0.272 (-1.302 to 0.757) | 0.604 | 1.053 (-0.265 to 2.371) | 0.117 | 0.727 (-0.028 to 1.482) | 0.059 | -0.128 (-0.876 to 0.620) | 0.737 |
| lag0-2 | -0.865(-1.883 to 0.153) | 0.096 | -0.603 (-1.618 to 0.413) | 0.245 | 0.522 (-0.936 to 1.979) | 0.483 | -1.358 (-3.286 to 0.569) | 0.167 | 0.013 (-1.051 to 1.078) | 0.981 | -0.842 (-1.816 to 0.131) | 0.090 |
| lag0-3 | -0.916 (-2.221 to 0.389) | 0.169 | -0.749 (-2.099 to 0.601) | 0.277 | -3.704 (-5.631 to -1.776) | 0.000 | 0.918 (-1.609 to 3.444) | 0.476 | -1.767(-3.147 to -0.388) | 0.012 | -0.132 (-1.287 to 1.025) | 0.824 |
| lag0-4 | 2.139 (0.551 to 3.727) | 0.008 | 1.494 (-0.116 to 3.104) | 0.069 | 3.737  (1.405 to 6.069) | 0.002 | 0.055 (-3.036 to 3.146) | 0.972 | 1.132 (-0.623 to 2.887) | 0.206 | -0.375 (-1.668 to 0.918) | 0.570 |
| lag0-5 | -2.781 (-4.509 to -1.054) | 0.002 | -2.190 (-3.977 to -0.403) | 0.016 | -5.545 (-8.137 to -2.953) | 0.000 | 1.472 (-2.015 to 4.958) | 0.408 | -2.149 (-4.150 to -0.147) | 0.035 | 0.048(-1.35 to 1.446) | 0.947 |
| lag0-6 | 4.658 (3.633 to 5.683) | 0.000 | 5.094 (3.985 to 6.204) | 0.000 | 9.968 (8.379 to 11.557 | 0.000 | 0.356 (-1.762 to 2.475) | 0.742 | 6.234 (4.974 to 7.494) | 0.000 | 6.073 (5.060 to 7.086) | 0.000 |
| Deep sleep duration/Light sleep duration | | | | | | | | | | | | |
| lag0 | -0.002 (-0.003 to -0.001) | 0.000 | -0.002 (-0.003 to -0.001) | 0.001 | -0.002 (-0.003 to -2.2E-04) | 0.026 | -0.001 (-0.003 to 0.001) | 0.327 | 7.22E-05(-0.001 to 0.001) | 0.897 | -0.002(-0.004 to -0.001) | 0.000 |
| lag0-1 | -0.001 (-0.003 to 0.001) | 0.338 | 1.35E-04(-0.002 to 0.002) | 0.899 | -0.001(-0.004 to 0.002) | 0.470 | -0.002 (-0.006 to 0.002) | 0.271 | -0.002 (-0.004 to  0.001) | 0.139 | -0.001 (-0.003 to 0.002) | 0.632 |
| lag0-2 | 0.003(-4.16E-05 to 0.006) | 0.053 | 0.001 (-0.002 to 0.004) | 0.574 | 0.002 (-0.002 to 0.006) | 0.357 | 0.006 (4.0E-4 to 0.011) | 0.035 | 7.25E-05(-0.003 to 0.003) | 0.964 | 0.002 (-0.001 to 0.005) | 0.203 |
| lag0-3 | -0.001 (-0.004 to 0.003) | 0.791 | -6.20E-05(-0.004 to 0.004) | 0.975 | 0.004 (-0.002 to 0.009) | 0.212 | -0.001 (-0.009 to 0.006) | 0.749 | 0.003(-0.001 to 0.007) | 0.169 | -0.001 (-0.005 to 0.002) | 0.467 |
| lag0-4 | -0.003 (-0.008 to 0.001) | 0.161 | -0.002 (-0.007 to 0.003) | 0.374 | -0.011 (-0.017 to -0.004) | 0.002 | -0.004(-0.013 to 0.005) | 0.404 | -0.004(-0.009 to 0.001) | 0.164 | -4.6E-04(-0.004 to 0.003) | 0.812 |
| lag0-5 | 0.009 (0.004 to 0.014) | 0.001 | 0.008 (0.002 to 0.013) | 0.004 | 0.019 (0.011 to 0.026) | 0.000 | 0.008 (-0.002 to 0.018) | 0.127 | 0.009 (0.003 to 0.0152) | 0.002 | 0.005 (0.001 to 0.009) | 0.012 |
| lag0-6 | -0.014 (-0.017 to -0.011) | 0.000 | -0.016 (-0.019 to -0.013) | 0.000 | -0.030 (-0.035 to -0.026) | 0.000 | -0.015 (-0.021 to -0.009) | 0.000 | -0.023 (-0.027 to -0.020) | 0.000 | -0.025 (-0.028 to -0.022) | 0.000 |
| Times of WASO per hour of sleep | | | | | | | | | | | | |
| lag0 | -0.001 (-0.002 to  3.16E-04) | 0.188 | -0.001 (-0.002 to  -1.4E-04) | 0.025 | -0.002 (-0.003 to -2.28E-04) | 0.024 | -0.001 (-0.003 to 4.5E-04) | 0.146 | -0.001 (-0.003 to  -2.0E-04) | 0.021 | -3.4E-04(-0.001 to 0.001) | 0.508 |
| lag0-1 | 4.88E-04(-0.001 to 0.002) | 0.618 | 2.02E-05(-0.002 to 0.002) | 0.984 | 0.001 (-0.001 to 0.004) | 0.357 | 0.001 (-0.002 to 0.005) | 0.517 | 0.001 (-0.001 to 0.004) | 0.287 | -0.001(-0.003 to 0.001) | 0.491 |
| lag0-2 | -3.32E-04(-0.003 to 0.002) | 0.815 | 0.001(-0.002 to 0.004) | 0.524 | -0.002 (-0.006 to 0.002) | 0.416 | -0.003 (-0.008 to 0.002) | 0.304 | -4.8E-04 (-0.004 to 0.003) | 0.783 | 0.002 (-0.001 to 0.004) | 0.229 |
| lag0-3 | -0.001 (-0.005 to 0.002) | 0.487 | -0.001 (-0.005 to 0.003) | 0.665 | 0.002(-0.003 to 0.007) | 0.484 | 0.002 (-0.005 to 0.008) | 0.641 | -0.004 (-0.008 to 0.0009) | 0.119 | -0.002(-0.005 to 0.001) | 0.199 |
| lag0-4 | 0.001 (-0.003 to 0.005) | 0.621 | -0.001 (-0.005 to 0.004) | 0.735 | -0.002 (-0.009 to 0.004) | 0.447 | 0.005(-0.004 to 0.013) | 0.281 | 0.006(-1.03E-04 to 0.011) | 0.054 | 0.001 (-0.003 to 0.004) | 0.673 |
| lag0-5 | 0.002 (-0.002 to 0.007) | 0.298 | 0.004 (-0.001 to 0.009) | 0.133 | 0.004(-0.003 to 0.011) | 0.285 | -0.006(-0.015 to 0.004) | 0.239 | -0.003 (-0.009 to 0.003) | 0.370 | 4.04E-04(-0.003 to 0.004) | 0.829 |
| lag0-6 | -0.004(-0.007 to -0.001) | 0.006 | -0.004 (-0.007 to -0.001) | 0.006 | -0.004 (-0.008 to 2.74E-04) | 0.067 | 0.007 (0.001 to 0.012) | 0.020 | 0.001 (-0.003 to 0.006) | 0.464 | -0.001(-0.004 to 0.001) | 0.365 |
| Duration of WASO/Total sleep duration | | | | | | | | | | | | |
| lag0 | -4.8E-05(-2.78E-04 to 1.81E-04) | 0.681 | -1.95E-04(-4.2E-04 to  3.20E-05) | 0.092 | -2.1E-04 (-0.001 to 1.32E-04) | 0.226 | -4.2E-04 (-0.001 to -1.0E-05) | 0.045 | -2.1E-04 (-4.5E-04 to 2.30E-05) | 0.077 | -1.69E-06(-2.4E-04 to 2.4E-04) | 0.989 |
| lag0-1 | -2.35E-04(-0.001 to 2.21E-04) | 0.313 | -1.0E-04 (-0.001 to 3.48E-04) | 0.657 | -2.9E-04 (-0.001 to 3.6E-04) | 0.384 | 0.001 (-2.9E-04 to 0.001) | 0.205 | -1.8E-04 (-0.001 to 2.97E-04) | 0.456 | -5.39E-04(-0.001 to -6.4E-05) | 0.026 |
| lag0-2 | 4.22E-04(-2.24E-04 to 0.001) | 0.200 | 3.63E-04 (-2.8E-04 to 0.001) | 0.270 | 0.001 (-2.42E-04 to 0.002) | 0.148 | -1.27E-04 (-0.001 to 0.001) | 0.838 | 3.78E-04 (-2.98E-04 to 0.001) | 0.273 | 7.6E-04 (1.46E-04 to 0.001) | 0.015 |
| lag0-3 | -1.97E-04(-0.001 to 0.001) | 0.641 | -1.1E-04 (-0.001 to 0.001) | 0.803 | -0.001 (-0.002 to 0.001) | 0.326 | -0.001 (-0.002 to 0.001) | 0.415 | 7.18E-05(-0.001 to 0.001) | 0.872 | 2.84E-05(-0.001 to 0.001) | 0.940 |
| lag0-4 | 1.80E-04(-0.001 to 0.001) | 0.727 | -2.52E-05(-0.001 to 0.001) | 0.961 | 0.001 (-0.001 to 0.002) | 0.301 | 4.89E-04 (-0.001 to  0.002) | 0.625 | -2.31E-04(-0.001 to 0.001) | 0.684 | -3.0E-04 (-0.001 to 4.74E-04) | 0.407 |
| lag0-5 | 3.76E-04(-0.001 to 0.001) | 0.501 | 0.001 (-0.001 to 0.002) | 0.324 | -0.001 (-0.002 to 0.001) | 0.373 | 0.002 (-4.39E-04 to 0.004) | 0.116 | 4.45E-04(-0.001 to 0.002) | 0.492 | 1.1E-04 (-0.001 to 0.001) | 0.805 |
| lag0-6 | -0.001 (-0.002 to -3.87E-04) | 0.002 | -0.001 (-0.002 to -0.001) | 0.000 | -4.51E-04(-0.001 to 0.001) | 0.381 | -0.002 (-0.003 to -4.38E-04) | 0.009 | -0.001 (-0.002 to -2.85E-04) | 0.008 | -0.001 (-0.002 to -4.7E-04) | 0.001 |

Note: data areβ (95%CI). β indicates partial regression coefficient. Estimates were associated with per 1-interquartile range increase in concentration of each pollutant. Adjusted for age, sex, BMI, city development level, altitude, year, month, and day of week.

Abbreviations: CI, confidence intervals; CO, carbon monoxide; NO_2_, nitrogen dioxide; O_3_, ozone; PM_2.5_, particulate matter with aerodynamic diameter ≤2.5 μm; PM_10_, particulate matter with aerodynamic diameter ≤10 μm; SO_2_, sulfur dioxide; WASO: wake after sleep onset.
